# Csk-homologous kinase (Chk) is an efficient inhibitor of Src-family kinases but a poor catalyst of phosphorylation of their C-terminal regulatory tyrosine

**DOI:** 10.1186/s12964-017-0186-x

**Published:** 2017-08-07

**Authors:** Gahana Advani, Ya Chee Lim, Bruno Catimel, Daisy Sio Seng Lio, Nadia L. Y. Ng, Anderly C. Chüeh, Mai Tran, Mohd Ishtiaq Anasir, Heather Verkade, Hong-Jian Zhu, Benjamin E. Turk, Thomas E. Smithgall, Ching-Seng Ang, Michael Griffin, Heung-Chin Cheng

**Affiliations:** 1 Department of Biochemistry & Molecular Biology, University of Melbourne, Parkville, VIC 3010 Australia; 2 Bio21 Biotechnology and Molecular Science Institute, University of Melbourne, Parkville, VIC 3010 Australia; 3 Cell Signalling Research Laboratories, School of Biomedical Sciences, University of Melbourne, Parkville, VIC 3010 Australia; 4 Walter and Eliza Hall Institute for Medical Research and Department of Medical Biology, University of Melbourne, Parkville, VIC 3010 Australia; 5 Department of Pharmacology, Yale University School of Medicine, New Haven, CT USA; 6 PAP Rashidah Sa’adatul Bolkiah Institute of Health Sciences, Universiti Brunei Darussalam, Gadong, Brunei Darussalam; 7 Department of Microbiology and Molecular Genetics, University of Pittsburgh School of Medicine, Pittsburgh, PA USA; 8 Department of Surgery, University of Melbourne, Royal Melbourne Hospital, Parkville, VIC 3052 Australia

**Keywords:** Src-family protein kinases, Tumor suppressor, Catalysis, colon cancer, C-terminal Src kinase, Csk, Chk

## Abstract

**Background:**

C-terminal Src kinase (Csk) and Csk-homologous kinase (Chk) are the major endogenous inhibitors of Src-family kinases (SFKs). They employ two mechanisms to inhibit SFKs. First, they phosphorylate the C-terminal tail tyrosine which stabilizes SFKs in a closed inactive conformation by engaging the SH2 domain in *cis*. Second, they employ a non-catalytic inhibitory mechanism involving direct binding of Csk and Chk to the active forms of SFKs that is independent of phosphorylation of their C-terminal tail. Csk and Chk are co-expressed in many cell types. Contributions of the two mechanisms towards the inhibitory activity of Csk and Chk are not fully clear. Furthermore, the determinants in Csk and Chk governing their inhibition of SFKs by the non-catalytic inhibitory mechanism are yet to be defined.

**Methods:**

We determined the contributions of the two mechanisms towards the inhibitory activity of Csk and Chk both in vitro and in transduced colorectal cancer cells. Specifically, we assayed the catalytic activities of Csk and Chk in phosphorylating a specific peptide substrate and a recombinant SFK member Src. We employed surface plasmon resonance spectroscopy to measure the kinetic parameters of binding of Csk, Chk and their mutants to a constitutively active mutant of the SFK member Hck. Finally, we determined the effects of expression of recombinant Chk on anchorage-independent growth and SFK catalytic activity in Chk-deficient colorectal cancer cells.

**Results:**

Our results revealed Csk as a robust enzyme catalysing phosphorylation of the C-terminal tail tyrosine of SFKs but a weak non-catalytic inhibitor of SFKs. In contrast, Chk is a poor catalyst of SFK tail phosphorylation but binds SFKs with high affinity, enabling it to efficiently inhibit SFKs with the non-catalytic inhibitory mechanism both in vitro and in transduced colorectal cancer cells. Further analyses mapped some of the determinants governing this non-catalytic inhibitory mechanism of Chk to its kinase domain.

**Conclusions:**

SFKs are activated by different upstream signals to adopt multiple active conformations in cells. SFKs adopting these conformations can effectively be constrained by the two complementary inhibitory mechanisms of Csk and Chk. Furthermore, the lack of this non-catalytic inhibitory mechanism accounts for SFK overactivation in the Chk-deficient colorectal cancer cells.

**Electronic supplementary material:**

The online version of this article (doi:10.1186/s12964-017-0186-x) contains supplementary material, which is available to authorized users.

## Background

Over-activation of Src-family tyrosine kinases (SFKs) contributes to the initiation and progression of many types of cancers such as colorectal carcinoma and leukemia [1–6]. In normal non-proliferating cells, SFKs are kept in the closed inactive conformation which is stabilised by two intramolecular inhibitory interactions: (i) binding of the SH3 domain to the SH2-kinase linker and (ii) binding of the C-terminal tail phosphotyrosine to the SH2 domain [7–11]. Phosphorylation of the C-terminal tail tyrosine is a pre-requisite for SFKs to adopt this closed inactive conformation. C-terminal Src kinase (Csk) and its homolog, Csk-Homologous Kinase (Chk) can specifically phosphorylate the C-terminal tail tyrosine of SFKs; they are therefore key endogenous inhibitors of SFKs in normal cells [12, 13]. In spite of the prevalence of over-activation of SFKs in cancer, oncogenic mutations of the *SFK* genes are rare (reviewed in [14, 15]), implying that their over-activation in cancer is caused by dysregulation of their upstream activators and inhibitors. Indeed, over-activation of SFKs in cancer cells has been found to be a result of one of the following contributing events: (i) upregulation of the phosphatases that target the inhibitory C-terminal tail phosphotyrosine [16, 17], (ii) reduced expression of Chk [18] and (iii) reduced expression of the scaffolding protein Cbp/PAG1 that directs co-localization of Csk and Chk with SFKs [19, 20].

Owing to their functional significance in constraining the oncogenic activity of SFKs, Csk and Chk are expressed at different levels in all mammalian cells. While Csk is ubiquitously expressed in all cells, Chk exhibits a much more restricted expression pattern. It is expressed at high levels in brain and haematopoietic cells [12, 21–25]. Additionally, it is also expressed in several other cell types and tissues such as colon tissue and smooth muscle cells [18, 26], albeit at lower levels [27].

The phenotypes of *Csk*- and *Chk*-deficient mice reveal significant functional redundancy of the two proteins when co-expressed in the same cells [28–30]. Presumably, the Csk and Chk performed overlapping functions by phosphorylating SFKs. Besides performing overlapping functions, Csk and Chk also perform specific functions. For example, Chk expression is suppressed in colorectal cancer cells and brain tumour cells [18, 31]; its deficiency contributes to over-activation of SFKs, uncontrolled growth, proliferation, and/or migration of the cancer cells [18, 31]. Although Csk is expressed in these cancer cells, it fails to suppress SFK activity and halt aberrant cell growth and proliferation. These pieces of evidence suggest that Chk performs specific functions in normal colon and brain cells, and Csk cannot compensate for its deficiency. The findings also imply that Chk is a potential tumour suppressor and its deficiency contributes to colorectal cancer and brain tumours.

Inactive SFKs adopting the closed conformation can be activated by three main mechanisms. The first mechanism involves dephosphorylation of the C-terminal tail tyrosine by phosphatases [16, 19, 32–37]. Both Csk and Chk can inhibit SFKs activated by this mechanism by phosphorylation of their C-terminal tail tyrosine. In this manuscript, we compare the efficiencies of Csk and Chk in phosphorylating and inhibiting the SFKs activated by this mechanism.

The second mechanism of SFK activation involves binding of protein regulators such as p130^Cas^ to the SH2 and SH3 domains [38–40], displacing one or both of the inhibitory intramolecular interactions in SFKs. The third mechanism of activation of SFKs is achieved by autophosphorylation of the consensus activating tyrosine in the activating loop of the kinase domain (reviewed in [41, 42]). SFKs activated by the second and third mechanisms remain active regardless of the phosphorylation status of the C-terminal tail tyrosine. Thus, both Csk and Chk are unable to inhibit SFKs in these active conformations simply by phosphorylating the C-terminal tail tyrosine. Our previous studies revealed that besides phosphorylation of the C-terminal tail tyrosine of SFKs, Chk can employ an alternative mechanism to inhibit SFKs with an efficiency not shared by Csk [13, 43]. This alternative inhibitory mechanism of Chk involves direct binding to SFKs. Since binding and inhibition by Chk occur even in the absence of phosphorylation of the C-terminal tail tyrosine of SFKs, this mechanism is referred to as the non-catalytic inhibitory mechanism.

We hypothesize that SFKs are over-activated in colorectal cancer and brain tumour cells because the inability of Csk to perform this non-catalytic inhibitory mechanism would prevent it from compensating for the Chk deficiency in these cells [18, 44]. In agreement with this hypothesis, our results show that even when active Csk is expressed in the Chk-deficient DLD1 colorectal cancer cells, Src is over-activated, suggesting that the co-expressed Csk cannot effectively constrain the activity of Src in these cancer cells. To further test the validity of this hypothesis, we examined the effects of induced expression of recombinant Chk in DLD1 cells on Src kinase activity. We found that recombinant Chk could effectively inhibit Src activity without significantly altering the phosphorylation level of its C-terminal tail tyrosine. Furthermore, we demonstrated that recombinant Chk could form complexes with Src in the transduced DLD1 cells. These results suggest that the recombinant Chk employed the non-catalytic inhibitory mechanism to suppress Src kinase activity. In the follow-up studies described in this manuscript, we sought to define the molecular basis of the non-catalytic inhibitory mechanism of Chk. We demonstrated that Chk could bind an activated SFK mutant with an affinity much higher than that of Csk, and Chk but not Csk could efficiently inhibit this SFK mutant with the non-catalytic inhibitory mechanism. We also mapped some of the major determinants governing the high affinity binding and inhibition of SFKs of Chk to its kinase domain.

## Methods

### Materials

The Csk/Chk optimal peptide substrate (KKKGESFEDQDEGIYWNVGPEA), two SFK-specific peptide substrates including Src-optimal peptide (AEEEIYGEFEAKKKK) and cdc2 (6–20) peptide (KVEKIGEGTYGVVKK) were synthesized as described previously [45, 46]. Lentiviral pLVX-Tight-puro and pLVX-Advance plasmids and were purchased from Clontech. The lentivirus packaging plasmid psPAX.2 and envelop plasmid pMD2.G were from Addgene. The HEK293FT cell line for generating the lentivirus, Opti-prep media and Lipofectamine 2000 were from Invitrogen. The Csk baculovirus was a gift from Dr. David Morgan, University of California, San Francisco [47]. The purified anti-Src monoclonal (Mab327) antibody was a generous gift from Dr. Donald Fujita, University of Calgary. The anti-Chk monoclonal antibody (Matk/Chk D216U rabbit mAb) was from Cell Signalling Technology. Expression and purification of Csk expressed in *Spodoptera frugiperda* 9 (*Sf9)* cells were described in our previous publications [43, 48]. Recombinant Chk with a C-terminal extension (GSGSHHHHHH) containing a linker (GSGS) and a poly-His tag, was generated and purified as described in our previous report [49]. The design and generation of the baculovirus directing expression of the Hck (2PA-YEEI) mutant in *Sf9* cells were described by Lerner et al. [39]. Its expression and purification were described in our previous publication [39, 43]. Additional file 1: Figure S1 shows the purity of Csk and Chk preparations used in the studies presented in this manuscript.

### Generation, expression and purification of recombinant Src and Src (K295M) mutant

The pMK-nSrc plasmid containing the cDNA encoding the wild type human neuronal Src was synthesised by Invitrogen Geneart [50] and was used as the template for the PCR reaction. The DNA encoding the recombinant truncated Src was amplified by PCR using primer 1 (ATCGGGATCCATGCATCATCATCATCATCATGATTACAAGGATGACGATGACAAGGGTTCTGGTTCGGCGGCGTGACAACATTCGTGG) as the forward primer and primer 2 (CGATGAATTCTCACAGATTCTCGCCGGGCTGGTAC) as the reverse primer. The underlined motifs are the *Bam*H1 and *Eco*R1 restriction site sequences located in primer 1 and primer 2, respectively. The resultant PCR product was cloned into the pBacPAK9 vector (BD Clontech) to generate the pBacPAK9-Src plasmid. This plasmid was used to produce the baculovirus directing expression of a truncated Src protein of which the unique domain (corresponding residues 1–82) was deleted and a segment of the sequence HHHHHHDYKDDDDKGSGS containing a poly-His-tag, a Flag tag and a GSGS linker was attached at the N-terminus (Additional files 1 and 2: Figures S1 and S2). This truncated Src protein is referred to as Src in this manuscript. To generate the baculovirus directing expression of the kinase-dead Src mutant (K295M), we used the pLVX-nSrc (K295M)-GFP plasmid which was produced for use in our previous study [50] as the template. The DNA encoding the truncated Src (K295M) was amplified by PCR using primer 1 and primer 2. The resultant pBacPAK9-Src (K295M) was used to generate the baculovirus. The resultant truncated Src (K295M) mutant lacking the unique domain and containing the segment (HHHHHHDYKDDDDKGSGS) at the N-terminus is referred to as Src (K295M) (Additional files 1 and 2: Figures S1 and S2).

### Generation, expression and purification of recombinant Chk and its mutants

Expression and purification of wild type Chk is described in our previous publication [13]. Single residue mutants of Chk carrying one of the five mutations, R276A, R278A, R280A, R382A and K387A were generated with the use of the QuikChange Site-Directed Mutagenesis Kit by Stratagene according to the manufacturer’s instructions. All Chk mutants were expressed in *Sf*9 cells and purified using procedures similar to those described in our previous publication [13].

### Generation, expression and purification of recombinant Csk-Chk chimera

The Csk-Chk chimera consists of the regulatory domains of Csk and the kinase domain of Chk. The nucleotide sequence of its SH3 and SH2 domains was derived from *Csk* corresponding to residues 1–181 of the chimera*.* The kinase domain was from *Chk* (residues 182–467 of chimera) (Additional file 3: Figure S7A). The nucleotide sequence of *Csk-Chk chimera* was designed based on GenScript’s OptimumGene™ Design system (Centennial Ave., Piscataway, NJ, U.S) to increase protein expression in the *Sf9* insect cells. A PreScission protease cleavage site (LEVLQGP) was added, followed by a GSGS linker and a 6xHis tag. The nucleotide sequence of the chimera was codon optimised for expression in *Sf*9 cells and was flanked by *Bam*H1 and *Eco*R1 restriction sites. The DNA was synthesised by Genscript in pUC57 plasmid. *Csk-Chk Chimera* was subcloned into the *pBacPAK9* vector for generation of the recombinant baculovirus that directs expression of Csk-Chk chimera in *Sf*9 cells.

Similar to wild-type Chk, the Csk-Chk chimera was purified with DEAE column chromatography, followed by Ni^2+^-NTA column chromatography. Unlike wild-type Chk, the Csk-Chk chimera did bind to the DEAE column. For purification of Csk-Chk chimera, the crude cell lysate was applied to a DEAE anion exchange column. After washing the column with Buffer A [20 mM Tris-HCl pH 7.0, 10% (*v*/v) glycerol, 10 mM β-glycerophosphate, 0.2 mg/ml benzamidine, 0.1 mg/ml PMSF and 2 mM β-mercaptoethanol], proteins bound to the column were first eluted with a linear gradient of 0–0.3 M NaCl in 400 ml of Buffer A followed by a linear gradient of 0.3–0.5 NaCl in 100 ml of Buffer A. Fractions containing the Csk-Chk chimera were pooled, adjusted to pH 8 (with 1 M Tris-HCl of pH 8.0) and loaded onto a 1-ml Ni^2+^-NTA column. After loading onto the Ni^2+^-NTA column, the column was washed and the bound proteins were eluted from the column.

### Determination of the secondary structure of the Csk-Chk chimera by circular dichroism (CD)

For CD analysis, peak protein fractions were pooled and the Csk-Chk chimera was applied onto a Superdex 200 HiLoad 16/60 Size Exclusion Column (SEC) (GE Healthcare) pre-equilibrated with Buffer B [20 mM Tris-HCl pH 8.0, 10% (*v*/v) glycerol, 10 mM β-glycerophosphate, 0.5 mM tris (2-carboxyethyl) phosphine (TCEP), 100 mM NaCl]. The sample was loaded and run through the SEC at the rate of 2 ml/min and collected in 1 ml fractions.

Circular dichroism (CD) spectra were recorded from the Csk-Chk chimera (0.3 μM) in a quartz cuvette (1 mm path length) within a temperature-controlled sample chamber attached to the AVIV 410SF CD spectrometer (Lakewood, NJ, USA). Spectra were collected from 250 to 200 nm at 25 °C, recording at a wavelength step of 1.0 nm, with a bandwidth of 1.0 nm and a scanning speed of 20 nm/min. The average reading time was 4 s and the settling time was 0.33 s. For secondary structure estimation, data were reduced to 1 data point per nm, and data between the range of 200 to 240 nm were fitted to a combination of α-helix, β-strand and random coil. This was done by comparison with over 40 reference spectra of proteins with varying secondary structures, using the neural network algorithm K2D2 [51].

### Peptide library screening

The sequence preference for Csk was determined by screening a positional-scanning peptide library as previously described for Chk [52]. The library used contained a set of 180 peptide mixtures with the general sequence G-A-X-X-X-X-X-Y-X-X-X-X-A-G-K-K (biotin), where X is an equimolar mixture of the 18 amino acids (excluding Tyr and Cys). Peptide mixtures (50 μM) were arrayed in 1536 well plates in 50 mM Tris, pH 7.5, 10 mM MgCl_2_, 1 mM MnCl_2_, 1 mM DTT, and 0.1% Tween 20, 2 L per well. Reactions were initiated by adding Csk (to 7.5 or 15 μg/mL) together with [γ-^33^P] ATP (to 50 μM at 0.03 μCi/mL). After incubation at 30 °C for 2 h, 200 nL aliquots were transferred to a streptavidin membrane (Promega), which was washed, dried and exposed to a phosphor screen. Radiolabel incorporation into peptides was quantified using QuantityOne software (BioRad). Data were background corrected and normalized such that the average value at a given position was set to 1. The heat map was generated in Microsoft Excel from log_2_ transformed normalized data.

### Kinase activity assay using peptide substrates

To measure the kinetic parameters of phosphorylation (catalytic efficiencies) of Csk and Chk towards the Csk/Chk optimal peptide, Csk or Chk (0.2 μM) was incubated with 0 to 500 μM of Csk/Chk-optimal peptide in the presence of assay buffer [20 mM Tris-HCl, 10 mM MgCl_2_, 1 mM MnCl_2_, 50 μM Na_3_VO_4_ and 25 mM HEPES (pH 7.4)] and 100 μM [γ-^32^P] ATP (specific radioactivity: ~200–500 cpm/pmol). The reaction was performed for 20 min at 30 °C and terminated by the addition of 15 μl of 50% (*v*/v) acetic acid. The amount of phosphate incorporated into the peptides was quantified by scintillation counting of the reaction mixture which was spotted onto phosphocellulose P81 filter paper. Prior to counting, the spotted phosphocellulose P81 filter paper was washed extensively with 5% (*v*/v) phosphoric acid to reduce background noise.

### Kinase activity assay using protein substrates

As Src is a bona fide substrate of Csk and Chk [13, 53], Src (K295M) was used to compare the efficiencies of both Csk and Chk in phosphorylating the conserved tyrosine at the C-terminal tail of SFKs. Chk or Csk (0.03 μM) was incubated with Src (K295M) (0 to 4.34 μM) in kinase assay buffer and 100 μM [γ-^32^P] ATP (specific radioactivity: ~ 1000 cpm/pmol) at 30 °C for 20 min. The reaction was terminated with 10 μl SDS-PAGE sample buffer. SDS-PAGE gel was used to separate the proteins and the extent of phosphorylation was detected by autoradiography and scintillation counting of the phosphorylated Src (K295M) protein bands excised from the gel.

### Inactivation and phosphorylation stoichiometry of SFKs by Chk, Csk and Chk mutants

The inhibition assay was divided into two main steps: (i) pre-incubation of the enzymes (Chk, Csk or chimera) with SFK (Src or Hck (2PA-YEEI) and (ii) measurement of SFK activity.

To determine inactivation of Src by Chk or Csk, Src (0.5 μM) was incubated with Chk (0 to 1.04 μM) or Csk (0 to 0.7 μM) in the presence of assay buffer and 250 μM [γ-^32^P] ATP (specific radioactivity: ~200–500 cpm/pmol) for 20 min at 30 °C. The reaction mixtures were aliquoted to determine kinase activity and phosphorylation stoichiometry. To determine kinase activity, the pre-incubated samples were diluted 20-fold and further incubated with 250 μM Src optimal peptide, assay buffer and 250 μM [γ-^32^P] ATP. The reactions were terminated and phosphorylation was determined as mentioned in the “kinase activity assay using peptide substrates” section. The phosphorylation stoichiometry of the remaining pre-incubated aliquot was determined by SDS-PAGE and scintillation counting as mentioned in the “kinase activity assay using protein substrates” section.

The inactivation of Hck (2PA-YEEI) by Csk, Chk, Chk point mutants and the chimera was determined similarly. Hck (2PA-YEEI) (0.5 μM) was incubated with a range of concentrations of each enzyme. The pre-incubation was performed at 4 °C and 500 μM of another SFK-specific substrate peptide, the cdc2 (6–20) peptide was used to determine residual enzymatic activity [54].

### Phosphopeptide mapping

Src alone (0.4 μM), Src (0.4 μM) with Chk (1.04 μM) or Csk (0.75 μM), Src (K295M) alone (0.4 μM) and with Csk (0.75 μM) were incubated with [γ-^32^P] ATP under conditions detailed in the previous section. The phosphoproteins were separated by SDS-PAGE, transferred to a nitrocellulose filter, and the bands corresponding to the radioactively phosphorylated Src were excised and processed for tryptic digestion. The tryptic fragments were separated and identified by two-dimensional thin layer electrophoresis/TLC procedures as described previously^*13*^. The tryptic digest of the C-terminal fragment of Src containing the Tyr-527 residue results in two fragments. The spots corresponding to Tyr-527 and Tyr-416 on the phosphopeptide maps were identified as described in our previous reports ^*(4, 13)*^.

### Surface plasmon resonance (SPR) spectroscopy

Csk and Chk proteins [0.5–2 mg/ml (approximately 4000 nM)] were analysed using Hck (2PA-YEEI) protein immobilised over a carboxymethyl dextran sensor chip [CMD 500 Xantec bioanalytics GmbH (Duesseldorf, Germany)] using a BIAcore 2000 system. Hck (2PA-YEEI) was immobilised through the primary amino groups (amino-terminus or lysine residues) using ethyl-N′-dimethylaminopropyl-carbodiimide (EDC) and N-hydroxysuccinimide (NHS) chemistry. The sensor surface was activated by injecting 90 μL of 0.2 M EDC/0.05 M NHS at 10 μL /min. Hck (2PA-YEEI) (100 μg/ml in 10 mM acetate buffer pH 4.0) was injected (30 μL at 10 μL/min) over the activated sensor surface. No covalently bound protein was desorbed by injecting 30 μL of 10 mM glycine pH 2.0 and the surface was blocked using 100 μl of 1 M ethanolamine pH 8.2 (injection at 10 μL/min). An immobilisation level of 6053 RU corresponding to approximately 6.0 ng/mm^2^ was obtained. A blank channel was derivatised under the same conditions and used as blank control.

Prior to biosensor analysis, the Chk and Csk proteins [0.5–2 mg/ml (approximately 4000 nM)] were dialyzed into HBS buffer (10 mM HEPES pH 7.4, 150 mM NaCl, 3.4 mM EDTA, 0.005% (*v*/v) Tween 20). Varying concentration of Chk (4.40 μM, 2.20 μM, 1.10 μM, 0.55 μM and 0.28 μM) and Csk (10.80 μM, 5.40 μM, 2.70 μM, 1.35 μM and 0.68 μM) were injected (30 μl) over immobilised Hck (2PA-YEEI) at a flow rate of 100 μL/min) for biosensor analysis. Dissociation was performed with HBS buffer at the same flow rate for 360 s. Residual bound antigen was eluted and the surface regenerated between injections using 40 μL glycine pH 2.0. This treatment did not denature the protein immobilised onto the sensor surface as shown by equivalent signals on reinjection of Chk and Csk proteins. Binding curves were analysed globally using a 1 to 1 L model that includes terms for mass transfer of analyte to the surface. SPR analyses were also conducted for other Chk mutants [Chk (R276A), Chk (R278A), Chk (R280A), Chk (R382A), Chk (K387A) and the Csk-Chk chimera] with varying concentrations. To enable a comparison of the binding activities of Csk, Chk and Chk mutants, their molecular binding activities (M.B.As) were calculated from the following equation [55]:

$$ \mathrm{M}.\mathrm{B}.\mathrm{A}.=\frac{\mathrm{Response}\ \mathrm{of}\ \mathrm{analyte}\times \mathrm{M}.\mathrm{W}.\mathrm{of}\ \mathrm{Immobilized}\ \mathrm{ligand}}{\mathrm{Amount}\ \mathrm{of}\ \mathrm{Immobilized}\ \mathrm{ligand}\times \mathrm{M}.\mathrm{W}.\mathrm{of}\ \mathrm{analyte}}, $$

where Csk, Chk and Chk mutants are the analytes and Hck (2PA-YEEI) is the immobilized ligand.

### Mass spectroscopy

Src alone (0.3 mg/ml), Src with Chk (0.20 μM) or Csk (0.03 μM), Src (K295M) alone and with Csk (0.03 μM) were incubated with 250 μM ATP under conditions detailed in the previous section. The proteins were separated by SDS-PAGE and subject to in-gel tryptic digestion as described previously [56]. Equal amounts of tryptic peptides were loaded onto a LTQ Orbitrap Elite (Thermo Scientific) with a nanoelectrospray interface coupled to an Ultimate 3000 RSLC nanosystem (Dionex). The nanoLC system was equipped with an Acclaim Pepmap nano-trap column (Dionex – C18, 100 Å, 75 μm × 2 cm) and an Acclaim Pepmap analytical column (Dionex C18, 2 μm, 100 Å, 75 μm × 15 cm). The peptide mix (2 μl) was loaded onto the trap column at an isocratic flow of 4 μl/min of 3% CH_3_CN containing 0.1% formic acid for 5 min before the enrichment column was switched in-line with the analytical column. The eluents used for the liquid chromatography were 0.1% (*v*/v) formic acid (solvent A) and 100% CH_3_CN/0.1% formic acid (*v*/v; solvent B). The following gradient was used: (i) 3% to 12% B for 1 min, (ii) 12% to 35% B in 20 min, (iii) 35% to 80% B in 2 min and (iv) maintained at 80% B for 2 min followed by equilibration at 3% B for 7 min before the next sample injection. The LTQ Orbitrap Elite mass spectrometer was operated in both data dependent and targeted mode with nano ESI spray voltage of +1.8 kV, capillary temperature of 250 °C and S-lens RF value of 60%. In both modes, spectra were acquired first in positive mode with full scan scanning from m/z 300–1650 in the FT mode at 240,000 resolution followed. In the data dependent mode, MS/MS was carried out by High Energy Collision dissociation (HCD) of the top 10 most abundant precursor ions. In the targeted mode, the masses 652.28 (2+) and 1359.28 (3+) corresponding to the phosphopeptides Tyr-416 (activation loop) and Tyr-527 (tail tyrosine), respectively were selected for HCD. Data analysis was carried out using Proteome Discoverer (Thermo Scientific version 1.4) with Mascot as the search engine (Matrix Science version 2.4 on the UniprotKB database) and Skyline [57]. Search parameters were trypsin with maximum of 2 missed cleavages, precursor mass tolerance of 20 ppm and fragment mass tolerance of 0.2 Da. Fixed modifications used are carbamidomethyl of cysteine and variable modifications are oxidation (M) and phosphorylation (S/T/Y). A targeted false discovery rate (FDR) of 1% was applied and identified peptides were further processed with the PhophoRS algorithm [58].

### Insertion of the GFP and Chk-GFP genes into the pLVX-tight-puro vector for lentivirus production

The human *Chk-GFP* gene directing the expression of Chk (Accession: NP_647612) with its C-terminus fused to a Gly-Ser-Gly-Ser linker and the enhanced green fluorescent protein (eGFP; Accession number: AAK15492) was synthesized by ThermoFisher-Geneart and inserted into the pMK-RQ vector. It was subcloned into the lentiviral vector pLVX-Tight-puro (Clontech) at the *BamH1* and *EcoRI* restriction sites. The gene encoding GFP was cloned in the lentiviral vector to be used as the control as described previously [50].

### Transfection of HEK293FT cells for lentivirus production

The HEK293FT cells, used for the production of lentivirus, were grown at 37 °C in DMEM complete media supplemented with 10% foetal calf serum, 2 mM L-glutamine, 1 mM sodium pyruvate, 1 mM non-essential amino acids and 100 U/ml penicillin–streptomycin and incubated in the presence of 5% CO_2_. They were passaged when they were >80% confluent.

Lentivirus directing expression of recombinant GFP, Chk-GFP and the Tet-controlled transcriptional activator were generated separately by transfection of HEK293FT cells. Prior to transfection, two mixtures were prepared. Mixture 1 contained pMD2.G (2.5 μg), pSpax2 (6.5 μg) and pLVX-tight puro plasmid containing the gene encoding GFP or Chk-GFP, or pLVX-Advanced plasmid containing the gene encoding the Tet-controlled transcriptional activator. Mixture 1 was then added to 1.5 ml of Opti-Mem 1 medium and mixed gently. Mixture 2 was prepared by combining Lipofectamine 2000 (35 μl) with 1.5 ml of Opti-Mem 1 medium and mixed gently. Mixtures 1 and 2 were gently mixed and incubated at room temperature for 20 min prior to transfection.

HEK293FT cells at 70% confluence were used for transfection. Briefly, the cells were washed with 3 × 10 ml of DMEM medium without antibiotics and foetal calf serum. 5 ml of DMEM medium without antibiotics and foetal calf serum was added to the washed cells prior to the addition of the Mixtures 1 and 2. Three to four hours after incubation at 37 °C in the cell culture incubator, 2 ml of DMEM medium with 10% foetal calf serum was added to the cells. The transfected cells were cultured for 3 days. Each day, the culture medium containing the lentivirus was collected and the 10 ml of fresh DMEM medium with 10% foetal calf serum was added to the cells. The cultured medium collected on all 3 days was combined and filtered with a sterile 0.22 μm filter and stored at 4 °C prior to be used for transduction of the DLD1 colon cancer cells.

### Transduction of DLD1 cells with lentivirus

Cultured DLD1 cells were transduced with (i) the lentivirus generated with the pLVX-Tet-on Advanced plasmid (Mixture 2) and (ii) the lentivirus generated with the pLVX-Tight-puro plasmid containing the gene encoding GFP, or Chk-GFP (Mixture 1). Growth media was replaced the next day and the transduced cells were selected by incubation with puromycin (5 μg/ml) and G418 (7 mg/ml) antibiotics for more than 7 days. In a separate experiment, we determined the minimal concentrations of puromycin and G418 that kill over 95% of the untransduced DLD1 cells to be 2.5 μg/ml and 3.5 mg/ml (data not shown). Thus, the concentrations of puromycin and G418 we used in the selection experiment would cause cell death of the untransduced DLD1 cells. Expression of recombinant Chk-GFP and GFP, induced by doxycycline (5 μg/ml), was monitored by confocal microscopy and Western blotting of the lysates of the transduced DLD1 cells with and without doxycycline treatment.

### Immunofluorescence staining

For adherent cells fixation, 12 mm round coverslips (Cat. No. G401–12, ProSciTech) were placed into the wells of a 24-well tissue culture plate and incubated with poly-D-lysine overnight at 37 °C in a humidified environment. DLD1 cells were plated at a density of 17,500 cells per well. Doxycycline was added after 24 h to induce the expression of the target proteins. 48 h after the treatment, cells were fixed in 3.7% formaldehyde (Cat. No. 11–0705, Sigma) for 10 min at room temperature, permeabilised in 0.2% Triton X-100 for 10 min and blocked with 1% bovine serum albumin (BSA) in PBS buffer for 1 h at room temperature. The nucleus of the cells were stained with 4′,6′-diamidino-2-phenylindole (DAPI) for 10 min and coverslips were mounted onto glass slides using fluorescence mounting media (Cat. No. P36961, ThermoFisher). Fluorescence of GFP or Chk-GFP was detected using Leica sp5 confocal microscope. Images were annotated using (Fiji Is Just) ImageJ software.

### Demonstration of Chk-GFP/Src complex formation in transduced DLD1 cells

We performed co-immunoprecipitation experiments to examine if Src and recombinant Chk-GFP could form complexes in the transduced DLD1 cells. ~10^7^ transduced DLD1 cells, before and after induction with doxycycline (5 mg/ml, 48 h of treatment) were lysed using lysis buffer [(50 mM Tris, 50 mM β glycerophosphate, 50 mM NaF, 50 mM NaCl, 2 mg/ml benzamidine, 1 mM EDTA, 1% Triton-X-100) supplemented with phosphotase, protease inhibitors and fresh 50 mM octyl β-D-glucopyranoside.]. Recombinant Chk-GFP was immunoprecipitated with GFP-trap magnetic beads (ChromoTek, Cat. Number: CT-gtm-20) prepared with anti-EGFP nanobody derived from alpaca covalently coupled to magnetic beads using the manufacturers protocol. After washing the beads extensively using dilution buffer (two washes with 10× diluted lysis buffer, one wash with dilution buffer containing 100 mM NaCl), proteins bound to the beads and proteins in the unbound fractions were analysed by Western blotting with the anti-Src and the anti-Chk antibodies.

Additionally, proteomics approach was also used to examine if Src could selectively bind to recombinant Chk-GFP in the transduced DLD1. In this approach, the transduced DLD1 cells expressing GFP upon induction with doxycycline (5 mg/ml, 48 h of treatment) were used as the negative control. In brief, GFP and Chk-GFP were immunoprecipitated from the crude lysate of the doxycycline-treated DLD1-GFP cells and the DLD1-Chk-GFP cells with GFP-trap magnetic beads/The beads were washed 2 × 1 ml lysis buffer, 4 × 1 ml lysis buffer with 0.5 M NaCl and 2 × 1 ml lysis buffer. Proteins bound to the beads were digested with trypsin. The tryptic digests of the anti-GFP immunoprecipitates derived from the DLD1-Chk-GFP and DLD1-GFP cells were processed by LC-MS/MS in LTQ Orbitrap Elite (Thermo Scientific) and analysed as described in the sub-section entitled “Mass Spectrometry”.

### Measurement of the mRNA levels of Chk, Csk and ActB in DLD1 colorectal cancer cells by quantitative PCR

Quantitative RT-PCR was performed as previously described [59]. Total RNA was extracted from exponentially growing cells using RNeasy Mini Kit (Qiagen) according to the manufacturer’s instructions. cDNA synthesis was performed using Transcriptor First Strand cDNA Synthesis Kit with random hexamer (Roche). qRT-PCR was carried out using Power SYBR Green PCR Master Mix (Life Technologies) on 7500 Fast Real-Time PCR System (Life Technologies) according to the manufacturer’s instructions. cDNA equivalent to 10 ng RNA was amplified with 75 nM forward and 75 nM reverse primers in 10 μL reaction. Dissociation curves were performed to confirm specific amplifications without primer dimer formation. Samples were also subjected to gel electrophoresis analysis to confirm that the PCR products were of expected size. Abundance of mRNA expression was determined using the comparative C_T_ method and expressed relative to ACTB expression. The following cycling conditions were used: 1 cycle of 95 °C for 10 min, 50 cycles of 95 °C for 15 s and 60 °C for 1 min. The following primers were used for the detection of mRNA expression: ACTB-F: 5′- CACCTTCACCGTTCCAGTTT-‘3; ACTB-R: 5’-GATGAGATTGGCATGGCTTT-3′; Chk/Matk-F: 5′-TCGTGTTGCATCTTCGTCAT-‘3; Chk/Matk-R: 5’-CACAGATCGGAGAGGGAGAG-‘3; Csk-F: 5’-CACAGATCGGAGAGGGAGAG-‘3; Csk-R: 5’-CTGACCGCATGGACCGT-‘3.

### Soft agar colony-formation assay

Soft agar colony-formation assay determines the ability of cancer cells to undergo anchorage-independent growth. Soft agar plates were prepared as described previously [60]. Briefly, 1% (*w*/*v*) analytical grade agar was first mixed with growth media prior to addition to the 12-well plates to form the bottom matrix. Cells were then suspended in 0.7% (*w*/*v*) analytical grade agar in growth media. The suspensions at the cell densities of ~2500 cells per well were added on top of the bottom matrix. Cells were fed twice a week with growth media. Colonies formed by anchorage-independent growth of the cancer cells, were visualized after 7–30 days by staining with 0.005% crystal violet. Both the numbers and sizes of the colonies were measured by ImageJ software. For statistical analyses, differences in colony numbers between the un-induced (−doxycycline) and induced (+doxycycline) samples were performed in triplicate and presented as means ± standard derivation (S.D.) using student’s t-test. *p*-value below 0.05 (*p* < 0.05) was considered as significant.

## Results

### The intrinsic catalytic activity of Csk is higher than that of Chk

**Fig. 1:**
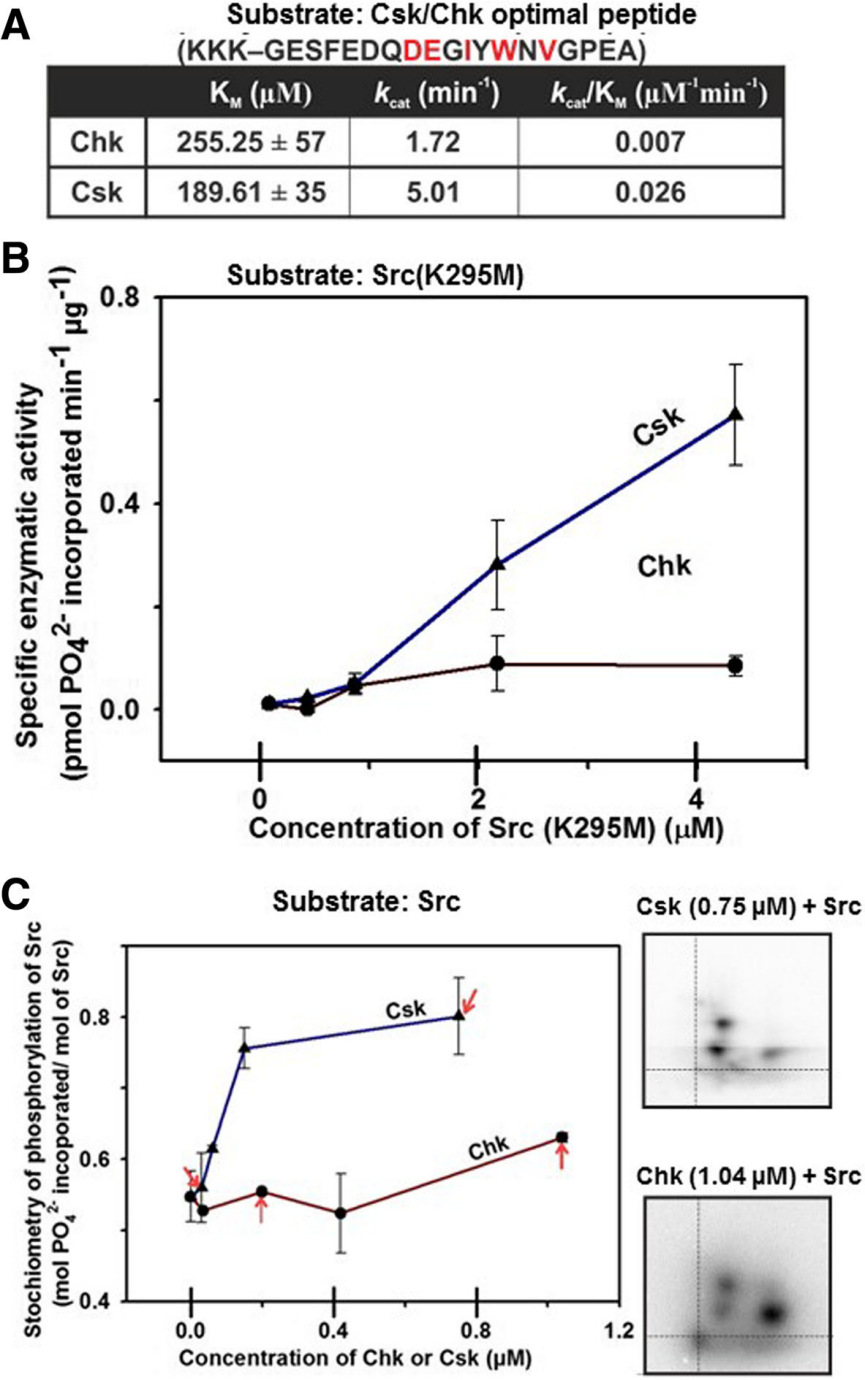
Csk and Chk exhibit different efficiencies in phosphorylating the Csk/Chk optimal peptide, Src (K295M) and Src. **a** Parameters of kinetic analysis of phosphorylation of Csk/Chk optimal peptide by Csk and Chk. The residues in *red font* were substrate specificity determinants of Chk and Csk identified by arrayed peptide library screen (Additional file 4: Figure S3). In the phosphorylation reaction, Csk or Chk (0.25 μM) was used to phosphorylate the substrate peptide at concentrations ranging from 0 to 0.5 mM. The initial velocities were plotted against the substrate peptide concentrations. The data points were fitted into Michaelis-Menten equation and transformed to Lineweaver-Burk plots (Additional file 4: Figure S3) to yield the V_max_ and K_M_ values. **b** Specific enzymatic activities of Csk and Chk in phosphorylation of the kinase-dead Src (K295M) mutant. Src (K295M) (0.08 μM to 4.3 μM) was incubated with Chk or Csk (0.03 μM), [γ-^32^P] ATP (250 μM) and assay buffer. The reaction mixtures containing Src (K295M) only were the negative controls. Gel slices containing the phosphorylated Src (K295M) were excised and [^32^P] phosphate associated with the slices was determined by scintillation counting for calculation of the specific enzymatic activities. **c** *Left panel*: Stoichiometry of phosphorylation of Src at both Tyr-416 and Tyr-527 in total in the presence and absence of increasing concentrations of Csk or Chk. The *red arrows* indicate the experimental data points corresponding to the phosphopeptide maps shown in the *right panels*. *Right panels*: The reaction mixtures contain Chk (1.04 μM) or Csk (0.75 μM), Src (0.6 μM) [γ-^32^P] ATP (250 μM) and assay buffer. Two-dimensional phosphopeptide maps of Src phosphorylated by Csk or Chk. The locations of the origin and phosphopeptides containing phospho-Tyr-416 or phospho-Tyr-527 in the maps are similar to those indicated in Additional file 6: Figure S5B

**Fig. 2:**
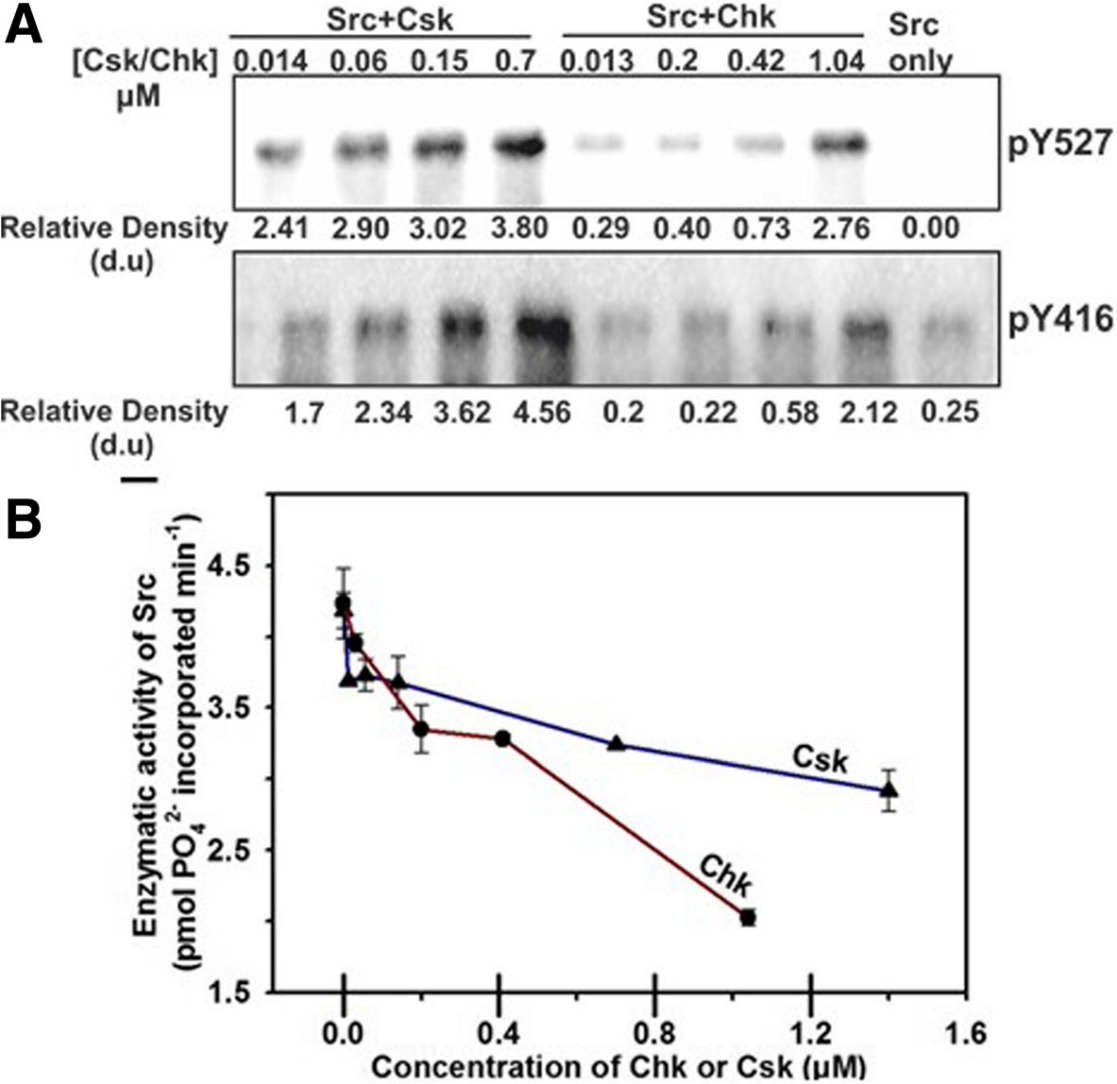
Csk and Chk exhibit different efficiencies in phosphorylating and inhibiting Src. **a** Western blot of phosphorylated Tyr-416 (lower) and Tyr-527 (upper) of Src in the presence of Csk (0.014–0.7 μM) or Chk (0.013–1.04 μM). Relative Densitometry units (d.u) corresponding to the two tyrosine phosphorylation sites are presented. The experiment was repeated three times. In all three replicates, Csk and Chk induced the same pattern of changes in Src phosphorylation at both sites. **b** Enzymatic activity of Src after incubation with varying concentrations of Csk or Chk. The enzymatic activities of the “Src alone” reaction mixtures were used as the controls

The efficiency of Csk and Chk in phosphorylating SFKs is governed in part by their intrinsic catalytic activities. However, SFKs are not suitable for use as in vitro substrates to measure their intrinsic catalytic activities. This is because Chk binds to SFKs tightly and does not phosphorylate them with Michaelis-Menten kinetics [13, 43]. We therefore sought to design a short synthetic peptide as the in vitro substrate. This peptide should contain common determinants recognised by the active sites of both enzymes. To this end, we employed the positional scanning peptide library screening approach [61] to search for this peptide. The results of this approach presented in Additional file 4: Figure S3 indicate that the peptide substrate preferences of Csk and Chk are very similar. Based upon these results, we define the optimal phosphorylation sequence of Csk and Chk as E-x-[Φ/E/D]-Y-Φ-x-Φ, of which x stands for any amino acid residue and Φ represents a hydrophobic amino acid residue (Additional file 4: Figure S3C). A consensus peptide substrate containing these major determinants was designed and synthesized [52]. This peptide, referred to as the Csk/Chk-optimal peptide, was used to determine the intrinsic catalytic activity of Csk and Chk (Fig. 1a). Phosphorylation of this peptide by both enzymes followed Michaelis-Menten kinetics (Additional file 5: Figure S4). Csk phosphorylated the peptide with a K_M_ value slightly lower than that of Chk, and with a *k*_*cat*_ value ~3-fold higher than that of Chk. The *k*_*cat*_/K_M_ ratio of Csk is 3.7-fold higher than that of Chk (Fig. 2a). These results indicate that the intrinsic catalytic activity of Csk is higher than that of Chk.

### Chk is a poor catalyst of C-terminal tail tyrosine phosphorylation but an efficient inhibitor of Src

We next determined the efficiencies of Csk and Chk in phosphorylating the C-terminal tail tyrosine of SFKs. To this end, we used Src and the kinase-dead Src (K295M) mutant (Additional files 1 and 2: Figures S1 and S2) as the in vitro substrates. Analysis by mass spectrometry revealed that Src (K295M) is phosphorylated by Csk at the C-terminal tail tyrosine (Tyr-527) (Additional files 3 and 6: Figures S5A and S7). Besides mass spectrometry, we also performed two-dimensional phosphopeptide mapping to compare the levels of phosphorylation at Tyr-416 and Tyr-527 in Src and Src (K295M) (Fig. 1c, right panels) [62]. In the presence of Csk, Src (K295M) was phosphorylated exclusively at Tyr-527, while Src was phosphorylated at Tyr-416 and Tyr-527 (Additional files 6 and 7: Figures S5 & S6). Phosphorylation at Tyr-416 presumably results from autophosphorylation of wild-type Src, because no phosphorylation of the activation loop tyrosine is observed when kinase-dead Src is incubated with Csk (Additional file 6: Figure S5B).

Figure 1b shows that Csk and Chk phosphorylated Tyr-527 of Src (K295M) with different efficiencies. At low Src (K295M) concentrations (0.08–0.72 μM), it was phosphorylated by Csk and Chk (0.03 μM) with similar efficiencies. However, at higher Src (K295M) concentrations (0.72–4.3 μM), Csk activity increased in proportion to the Src (K295M) concentration, while Chk activity remained the same. We previously demonstrated that Chk binds SFKs tightly [13, 43]. The tight binding of Chk to Src (K295M) likely limited dissociation of the phosphorylated Src (K295M) from Chk. Because of this anomalous property of Chk, it exhibited a much lower catalytic activity at the Src (K295M) concentrations used in our analysis (0.72–4.3 μM).

We next compared the efficiencies of Csk and Chk in phosphorylating Tyr-527 of wild type Src, and their effects in regulating the activity and Tyr-416 autophosphorylation of Src. Figure 1c revealed that incubation of Src with Csk (0.014 μM – 0.7 μM) led to a significant increase in the total level of phosphorylation of Src. Unlike Csk, incubation of Src with Chk did not significantly change the stoichiometry of phosphorylation of Src. At Chk concentrations ranging from 0.013 μM to 0.42 μM, there is no increase in stoichiometry of Src phosphorylation. As Chk concentration increased from 0.42 μM to 1.04 μM, the 2½ -fold increase in Chk concentration resulted in only a modest increase in phosphorylation stoichiometry of Src.

Two-dimensional phosphopeptide mapping and mass spectrometry analysis revealed that Src was phosphorylated at both Tyr-416 and Tyr-527 in the presence of Csk or Chk (Fig. 1c and Additional files 3, 6 and 7: Figures S5, S6 and S7). We then performed Western blot analysis using the anti-pY416 and anti-pY527 antibodies to monitor the changes in Src phosphorylation at both sites upon incubation with varying concentrations of Csk or Chk. The data indicate that Csk phosphorylated Tyr-527 of Src with a much higher efficiency than that of Chk (Fig. 2a). In agreement with our previous observation [13], both Chk and Csk facilitated autophosphorylation of Src at Tyr-416 (Fig. 2a). Although our results did not reveal the mechanism for this counterintuitive observation, the report by Wang et al. that Src undergoes dimerization in vitro may offer an explanation [63]. Presumably, Csk or Chk binding facilitates Src dimerization and in turn autophosphorylation *in trans* under these experimental conditions.

To compare the impacts of Csk and Chk on the catalytic activity of Src, we monitored the kinase activities of Src autophosphorylated alone and Src phosphorylated in the presence of Csk or Chk. Figure 2 shows that even though Chk phosphorylated Tyr-527 of Src to a level much lower than that of Csk, it was a much more efficient inhibitor of Src kinase activity. Furthermore, our data show that Chk could effectively suppress Src activity regardless of the level of autophosphorylation of Src at Tyr-416 (Fig. 2). These results suggest that Chk inhibited Src with a mechanism independent of Tyr-527 phosphorylation. Thus, our observations are in agreement with our previous findings that Chk can employ a non-catalytic, C-terminal tail tyrosine-independent inhibitory mechanism to suppress the activity of SFKs [13, 43].

### Chk exhibits a higher efficiency than Csk to inhibit SFKs by the non-catalytic inhibitory mechanism

**Fig. 3:**
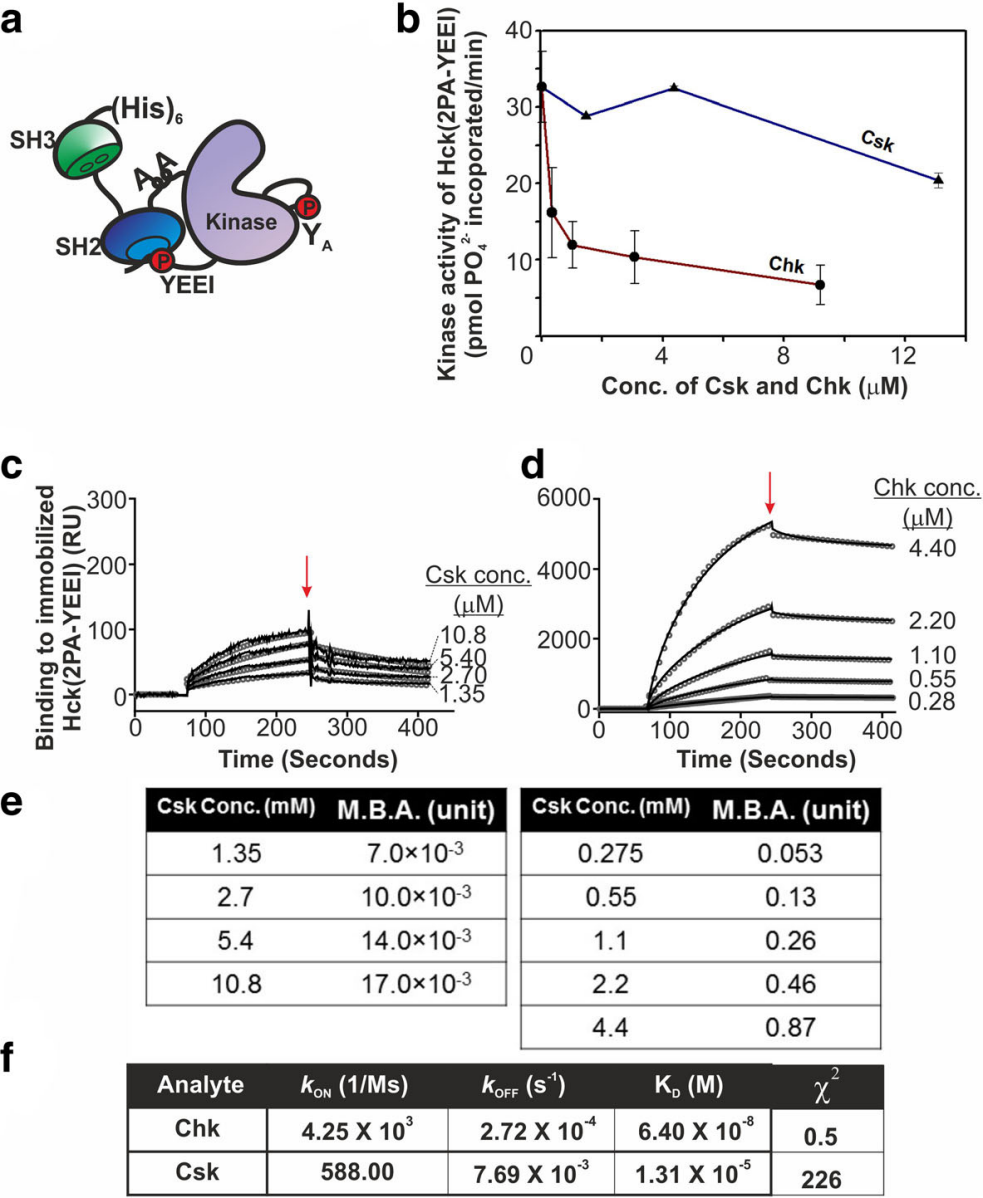
Chk but not Csk efficiently inhibits and tightly binds Hck (2PA-YEEI). **a** A schematic representation of the possible conformation adopted by Hck (2PA-YEEI) revealed in our previous studies [39]. The mutant is constitutively phosphorylated at the C-terminal YEEI motif. Upon phosphorylation, it binds tightly with the SH2 domain. The 2PA mutations which replace the two key proline residues in the SH2-kinase linker with alanine prevent the linker from binding to the SH3 domain to adopt the closed inactive conformation. Consequently, the mutant is autophosphorylated at consensus site (Y_A_) in the activation loop and remains constitutively active. **b** Enzymatic activity of Hck (2PA-YEEI) in the presence and absence of varying concentrations of Chk or Csk. **c-d** Sensorgrams of surface plasmon resonance spectroscopy showing the kinetics of interactions of Csk (**c**) and Chk (**d**) with Hck (2PA-YEEI) immobilised on sensor chips. Biosensor response is recorded as response units (RU). 100 μg/ml of Hck (2PA-YEEI) was immobilised onto the sensorchips. Designated concentrations of Csk and Chk were allowed to flow through the respective channels at a rate of 10 μl/min. At 250 s (*red arrow*), HBS buffer was injected to initiate dissociation of the protein complexes formed by Csk and Hck (2PA-YEEI) or Chk and Hck (2PA-YEEI). **e** Molecular binding activities (M.B.As) of Csk and Chk at all concentrations determined from the responses of Csk and Chk binding to the immobilized Hck (2PA-YEEI), and the amount of Hck (2PA-YEEI) immobilized on the sensor chip. **f** The association and dissociation rate constants (*k*_ON_ and *k*_OFF_) and equilibrium constants of dissociation (K_D_) of interactions between Csk or Chk with the immobilized Hck (2PA-YEEI)

Since wild type Src can be inhibited by both Tyr-527 phosphorylation and the non-catalytic inhibitory mechanism, it is not suitable for quantitative analysis to compare the efficiencies of the non-catalytic inhibitory mechanism of Csk and Chk. To overcome this technical problem, we used a constitutively active SFK mutant which we previously developed for our analysis [39]. This mutant, referred to as Hck (2PA-YEEI) is derived from the Src-family member hematopoietic cell kinase (Hck). Replacement of the C-terminal YQQQP motif with the YEEIP sequence permits the C-terminal tail of this Hck mutant to undergo robust autophosphorylation and phosphorylation by a Csk-like enzyme in cells [39]. Upon phosphorylation, the resultant pYEEI motif resembles the high affinity binding sequence of SFK SH2 domains [64, 65] (Fig. 3a). Consequently, it binds intramolecularly to the SH2 domain with an affinity ~150-fold higher than that of the C-terminal pYQQQ motif of wild type Hck [65]. The “2PA” mutation of Hck (2PA-YEEI) replaces the two conserved proline residues in the SH2-kinase linker with alanine. This mutated linker cannot bind the SH3 domain, preventing the Hck mutant from adopting the closed inactive conformation (Fig. 3a). Thus, even though the C-terminal tail tyrosine is constitutively phosphorylated and tightly bound to the SH2 domain, Hck (2PA-YEEI) is active (Fig. 3b) and autophosphorylated at the conserved activation loop tyrosine (Tyr-411 of Hck, homologous to Tyr-416 of Src) [39]. As such, Csk and Chk can only employ the non-catalytic inhibitory mechanism to inhibit Hck (2PA-YEEI).

As shown in Fig. 3b, Csk exhibited weak inhibitory activity towards Hck (2PA-YEEI) (0.5 μM) only at the highest concentration (13 μM) used in the assay. In contrast, Chk robustly suppressed the activity of Hck (2PA-YEEI) at all concentrations used in the experiment. Chk at 1.5 μM suppressed more than 60% of the activity of Hck (2PA-YEEI). The extent of inhibition reached 80% when Hck (2PA-YEEI) was incubated with 8.3 μM of Chk. These results indicate that Chk can effectively employ the non-catalytic inhibitory mechanism to inhibit Hck (2PA-YEEI) activity.

### Chk binds Hck (2PA-YEEI) with an affinity much higher than that of Csk

Since inhibition of SFKs by the non-catalytic inhibitory mechanism relies on direct binding of Csk and Chk to SFKs [13, 43], we performed surface plasmon resonance (SPR) studies to measure the kinetics of Csk and Chk binding to Hck (2PA-YEEI) immobilized on a sensor chip. Figure 3c and d show the sensorgrams of this binding of Csk and Chk to the immobilized Hck (2PA-YEEI), respectively. From the maximum response signals caused by binding of the analytes (Csk and Chk) in each sensorgram, we calculated the molecular binding activities (MBAs) of Csk and Chk (Fig. 3e and Additional file 8: Table S1). The MBA values [with units of mol of analyte bound per mol of immobilized Hck (2PA-YEEI)] reflect the proportions of immobilized Hck (2PA-YEEI) that participated in binding to Csk and Chk. Figure 3c-e show that Chk bound to the immobilized Hck (2PA-YEEI) with MBA values much higher than those of Csk. For example, when Csk and Chk of comparable concentrations (2.7 μM and 2.2 μM, respectively) were used in the analysis, the MBA value of Chk binding (0.46 unit) was ~46-fold higher than that of Csk binding (10 × 10^−3^ unit). These results suggest that a much higher proportion of Chk molecules participated in binding to the immobilized Hck (2PA-YEEI) compared to that of the Csk molecules.

Comparison of the kinetic parameters of Csk and Chk binding to the immobilized Hck (2PA-YEEI) (Fig. 3) revealed that the K_D_ of Csk (13.1 μM) is 205-fold higher than that of Chk (64.0 nM), indicating that Chk binds Hck (2PA-YEEI) with an affinity much higher that of Csk. The higher affinity of Chk is the result of a higher association rate constant (*k*_ON_) (4250 M^−1^ s^−1^ for Chk versus 588 M^−1^ s^−1^ for Csk, and a slower dissociation rate constant (*k*_OFF_) (0.27 × 10^−3^ s^−1^ for Chk versus 7.7 × 10^−3^ s^−1^ for Csk). Taken together, the data shown in Fig. 3 suggest that Chk binds SFKs with affinities much higher than those of Csk. The ability of Chk to efficiently inhibit SFKs with the non-catalytic inhibitory mechanism is likely attributed to its high affinity of binding to the active form of SFKs.

### The kinase domain of Chk contains the determinants governing tight binding and non-catalytic inhibition of SFKs

**Fig. 4:**
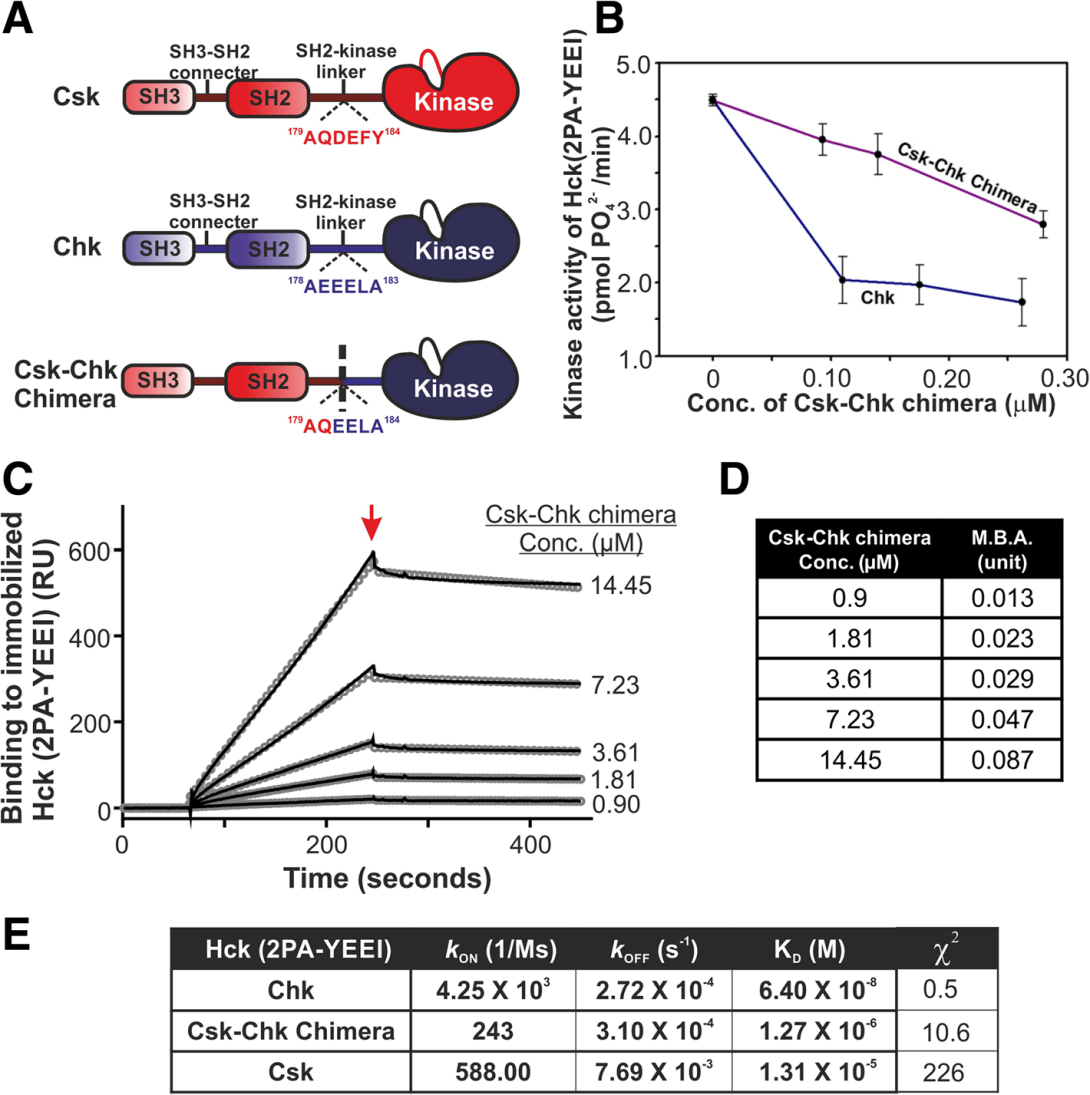
The major determinants governing high affinity binding and non-catalytic inhibition of SFKs reside in the segment containing the kinase domain of Chk. **a** A schematic illustration depicting the arrangements of functional domains in Csk, Chk and the Csk-Chk chimera. The *black dotted line* on the SH2-kinase linker of Csk-Chk chimera denotes the junction joining the sequences of Csk and Chk. The sequence at the junction of the chimera as well as those at the corresponding segment of Csk and Chk are shown. **b** Enzymatic activity of Hck (2PA-YEEI) in the presence and absence of the designated concentrations of Chk or Csk-Chk chimera. **c** Sensorgrams showing the kinetics of interactions of Csk-Chk chimera with the immobilized Hck (2PA-YEEI). The *red arrow* indicates the point when buffer was introduced to initiate dissociation of Csk-Chk chimera from the complex formed by Csk-Chk chimera and the immobilized Hck (2PA-YEEI). **d** Molecular binding activities of Csk-Chk chimera at the designated concentrations. **e** Comparison of the association rate constant (*k*_*a*_), dissociation rate constant (*k*_*d*_) and equilibrium constants of dissociation (K_D_) of Chk, Csk-Chk chimera and Csk. The kinetic parameters of binding of Csk and Chk to Hck (2PA-YEEI) are also shown in Fig. 3f

Reports by Lee et al. and Levinson et al. indicate that the motifs mediating binding of Csk and Src reside in their kinase domains [66–68]. Given the high degree of sequence homology of the kinase domains of Csk and Chk, we hypothesize that the kinase domain of Chk also contains the SFK-binding motifs [69]. To test this hypothesis, we first generated the recombinant Chk kinase domain and demonstrated that it could co-immunoprecipitate with SFKs [43]. However, the yield of recombinant Chk kinase domain was too low for further biochemical and biophysical studies. To circumvent this technical difficulty, we designed a Csk-Chk chimera in which the two regulatory domains (SH2 and SH3) of Csk were fused with the kinase domain of Chk (Fig. 4a, Additional file 9: Figure S8A).

Recombinant Csk-Chk chimera was expressed at a high level in *Sf*9 cells, and it could be purified to near homogeneity by sequential anion exchange, Ni^2+^-NTA and size-exclusion column chromatography (Additional file 9: Figure S8B). Circular dichroism analysis showed that the purified Csk-Chk chimera was folded (Additional file 9: Figure S8C). It phosphorylated the Csk/Chk optimal peptide with kinetic parameters similar to those of Chk (Additional file 9: Figure S8D), suggesting that replacement of the N-terminal segment containing the SH3 and SH2 domains of Chk with that of Csk did not adversely impact the intrinsic catalytic activity of Chk.

The non-catalytic inhibitory activity of Chk does not involve binding of Chk active site to the C-terminal tail tyrosine of Chk. Hck (2PA-YEEI) is suitable for the investigation to define the structural motifs in Chk governing its non-catalytic inhibitory activity because its C-terminal tail pYEEIP motif is tightly bound to the SH2 domain and therefore unavailable for binding to the Chk active site. Figure 4b shows that the Csk-Chk chimera could inhibit Hck (2PA-YEEI), albeit with an efficiency lower than that of Chk, indicating that the kinase domain of Chk retains some of the key determinants governing non-catalytic inhibition of SFKs. Analysis of the sensorgram of Csk-Chk chimera binding to immobilized Hck (2PA-YEEI) revealed that it bound Hck (2PA-YEEI) with MBA values lower than those of wild type Chk but higher than those of Csk (Figs. 3e, 4c and d and Additional file 10: Figure S9). This indicates that in comparison to Csk, a much higher proportion of Csk-Chk chimera participated in binding Hck (2PA-YEEI). Analysis of the kinetics of Csk-Chk chimera interaction with Hck (2PA-YEEI) revealed that it bound Hck (2PA-YEEI) with an association rate constant (*k*_ON_) (243 M^−1^ s^−1^) of the same order of magnitude as that of Csk (588 M^−1^ s^−1^_),_ but much lower than that of Chk (4.25 × 10^3^ M^−1^ s^−1^) (Fig. 4e). The rate of its dissociation (*k*_OFF_) from Hck (2PA-YEEI) is similar to that of Chk (3.1 × 10^−4^ s^−1^ for Csk-Chk chimera versus 2.72 × 10^−4^ s^−1^ for Chk). These results suggest that similar to Csk, Csk-Chk chimera slowly associates with Hck (2PA-YEEI) to form protein complexes. Once the complexes are formed, Csk-Chk chimera remains tightly bound to Hck (2PA-YEEI) because its *k*_OFF_ is as low as that of Chk. Since *k*_ON_ of the Csk-Chk chimera is much lower than that of Chk, the data indicate that replacement of the SH3 and SH2 domains of Chk with those of Csk reduces the rate of association. Presumably, appropriate intramolecular interactions of the SH3 and SH2 domains with the kinase domain of Chk facilitate Chk to adopt the configuration that rapidly associates with Src; these interactions are perturbed in the Csk/Chk chimera because the SH3 and SH2 domains of Chk are replaced by those of Csk.

In comparison with Csk binding and inhibition of Hck (2PA-YEEI) (Figs. 3b, 4b and e), the significantly higher MBA values and the significantly lower *k*_OFF_ value of Csk-Chk chimera account for its much higher efficiency in inhibiting Hck (2PA-YEEI) than that of Csk. Based upon our results, we conclude that the Chk kinase domain contains determinants governing its tight binding and inhibition of SFKs by the non-catalytic inhibitory mechanism.

### Point mutations of the five conserved basic residues in the αD-helix and αF-αG loop of Chk have little or no impact on binding and inhibition of Hck (2PA-YEEI)

Next, we sought to define the determinants governing tight binding and the non-catalytic inhibitory mechanism of Chk. Given the high degree of sequence similarity between the kinase domains of Csk and Chk, we explored the structure of the Csk/Src complex [66] for clues to elucidate the identities of these determinants (Additional file 11: Figure S10). The structure revealed that Csk interacts with Src by hydrogen bonds and electrostatic interactions at an interface of 1200 Å^2^ formed by the αD helix and αF-αG loop of Csk and αH-αI loop and αI/αI’ helices of the Src kinase domain. Levinson et al. discovered that five highly conserved basic residues in the αD helix and αF-αG loop of Csk (Additional file 11: Figure S10B) play significant roles in the binding of Csk to Src and efficient phosphorylation of Tyr-527 of Src by Csk. They showed that replacement of any one of the basic residues either completely abolished or significantly reduced the ability of Csk to bind and phosphorylate Src [66]. We hypothesized that the homologous basic residues in Chk including Arg-276, Arg-278 and Arg-280 of the αD helix and Arg-382 and Lys-387 of the αF-αG loop (Additional file 11: Figure S10) are critical to Chk binding and/or inhibition of SFKs. To assess the contribution of each of these residues to tight binding of SFKs and the non-catalytic inhibitory mechanism of Chk, we generated five Chk mutants carrying single point mutations that replace each of the residues with alanine for biochemical analyses (Additional file 11: Figure S10C).

Kinetic analyses of phosphorylation of the Csk/Chk-optimal peptide by the mutants revealed that the *k*_*cat*_/K_M_ value of wild type Chk is 1.5- to 11-fold higher than those of the Chk mutants (Additional file 12: Figure S11), suggesting that the mutations cause structural perturbations that reduce the intrinsic catalytic activity.

**Fig. 5:**
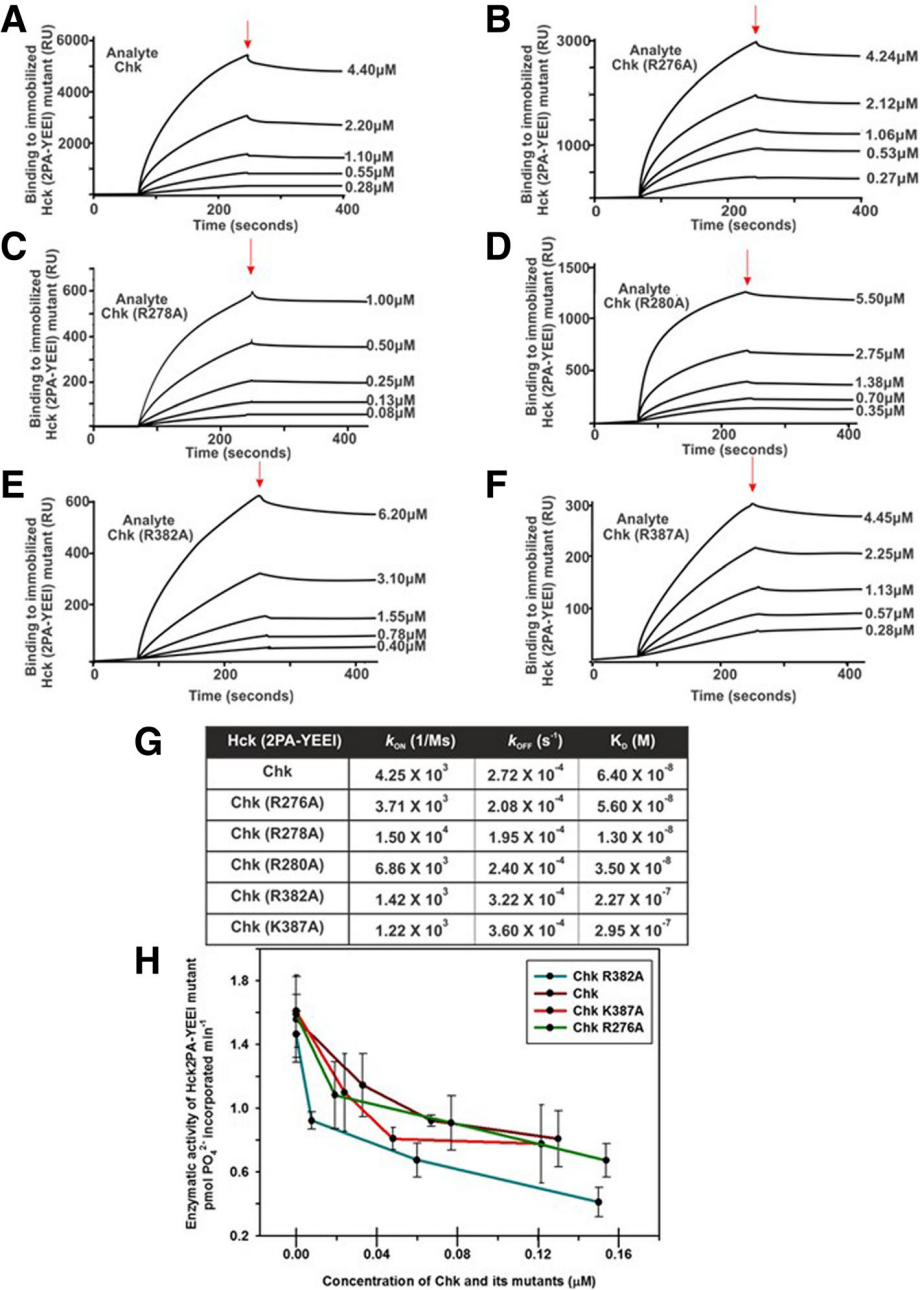
Effects of mutation of the conserved basic residues in the αD-helix and αF-αG loop region of Chk on its inhibition and binding of Hck (2PA-YEEI). **a-f** Sensorgrams depicting the kinetics of interactions of Chk and Chk mutants including Chk (R276A), Chk (R278A), Chk (R280A), Chk (R382A), Chk (K387A) with the immobilised Hck (2PA-YEEI). **g** Kinetic parameters of the interactions. **h** Enzymatic activities of Hck (2PA-YEEI) in the presence and absence of Chk or the Chk mutants [Chk (R276A), Chk (R382A) and Chk (K387A)] at the designated concentrations

Unlike the significant negative impact of point mutations of the conserved basic residues in the αD-helix of Csk on its affinity for Src observed by Levinson et al. [66], point mutations of the homologous residues in Chk had little or no impact on their binding affinity for SFKs (Fig. 5). The R276A, R278A and R280A mutations did not reduce the affinity of Chk for Hck (2PA-YEEI) (Fig. 5). However, point mutations R382A and K387A mapped to the αF-αG loop of Chk increase the K_D_ value by 4- to 5-fold (Fig. 5g). In spite of the increase, the K_D_ values (2.27 × 10^−7^ M and 2.95 × 10^−7^ M) of these mutants are still two orders of magnitude lower than that (1.30 × 10^−5^ M) of Chk. It is noteworthy that the increase in the K_D_ value is mainly a result of significant reduction in the rate of association (*k*_ON_); the mutations did not significantly alter the rate of dissociation (*k*_OFF_) (Fig. 5g).

Since the R382A and K387A mutations reduce the affinity of Chk for Hck (2PA-YEEI), we next examined if the mutations also impacted the ability of Chk to inhibit SFKs by the non-catalytic inhibitory mechanism. Figure 5h shows that they did not reduce the ability of Chk to inhibit Hck (2PA-YEEI). Thus, under the conditions we performed the experiments, the Chk mutants could still effectively bind to Hck (2PA-YEEI) to form complexes even when the *k*_ON_ values were reduced by ~3-fold. Once the protein complexes were formed, the slow rates of dissociation (*k*_OFF_) allowed the Chk mutants to remain bound to Hck (2PA-YEEI) to inhibit its activity. To further define the contributions of the basic residues in governing the non-catalytic inhibitory mechanism of Chk, we attempted to generate the 3A–Chk mutant in which Arg-276, Arg-278 and Arg-280 of the αD-helix is mutated to alanine and the 2A–Chk mutant in which Arg-382 and Lys-387 in the αF-αG loop were mutated to alanine. However, the yields of both mutants expressed in insect cells were very low and rapidly lost their kinase activity during purification (data not shown), suggesting that mutations of multiple basic residues significantly perturb the stability of Chk. Because of this technical problem, we were unable to conduct biochemical analyses of these two mutants. Nonetheless, our results demonstrated that single point mutations of the five conserved basic residues of Chk had no or mild impacts on its SFK-binding affinity and the ability to inhibit SFKs with the non-catalytic inhibitory mechanism.

### Recombinant Chk-GFP could inhibit and form protein complexes with Src in DLD1 colorectal cancer cells

**Fig. 6:**
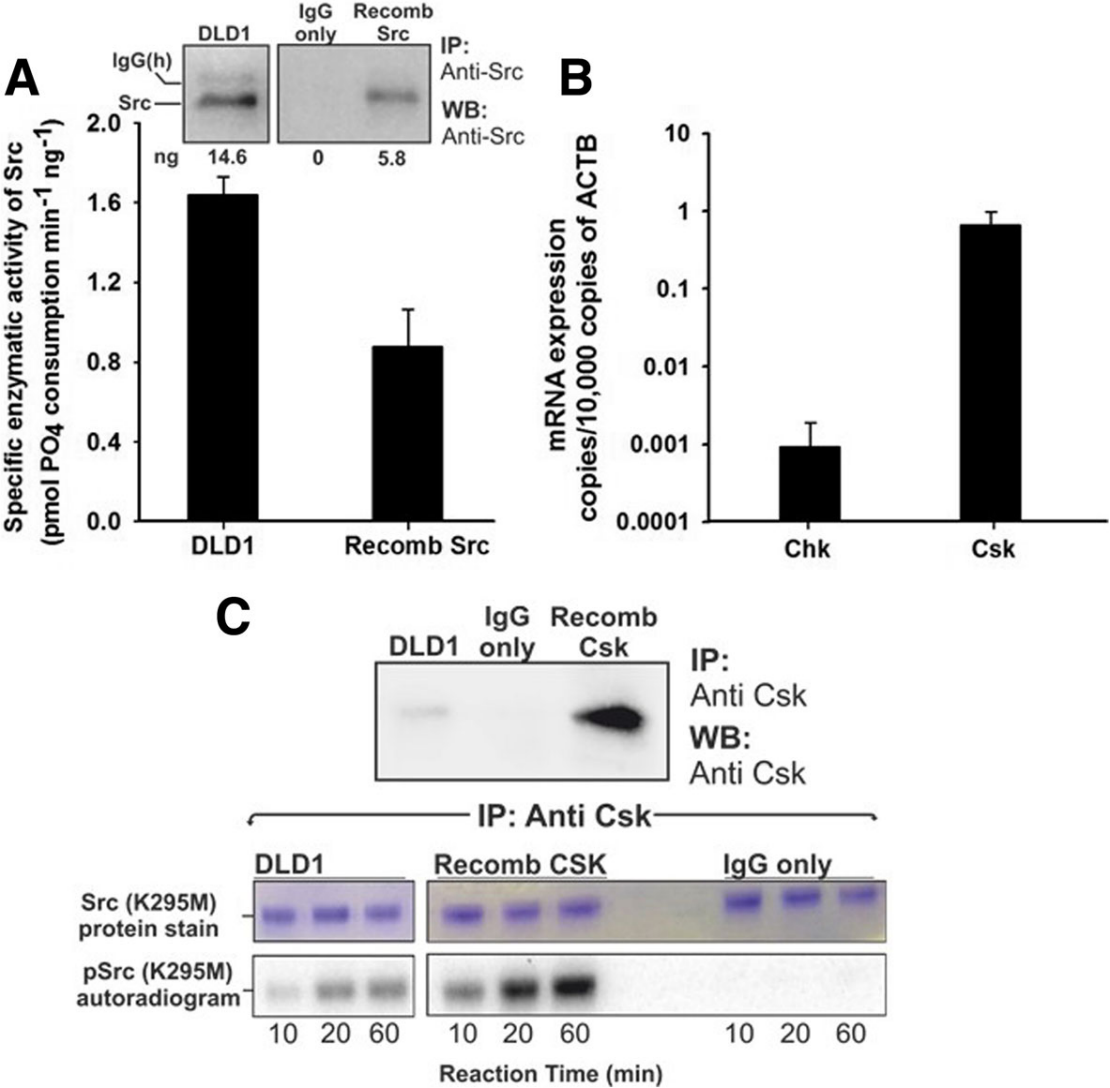
Src is over-activated in DLD1 colorectal cancer cells even though the co-expressed Csk is active. **a** Comparison of the specific enzymatic activity of Src immunoprecipitated from DLD1 cell lysate with that of recombinant unphosphorylated Src. Src was immunoprecipitated from DLD1 cells and its specific enzymatic activity was monitored using Src optimal peptide as the substrate. Recombinant unphosphorylated Src was used as the control. **b** The mRNA levels of *Csk* and *Chk* determined by qPCR. **c** Demonstration of phosphorylation of recombinant kinase-dead Src by Csk isolated from DLD1 cells. Csk was immunoprecipitated from the lysate of DLD1 cells and its activity was monitored using Src (K295M) as the substrate. The immunoprecipitated recombinant Csk was used as the control. The presence of Csk in the immunoprecipitates was monitored by Western blotting (*upper panel*). Src (K295M) (0.25 μM) was incubated with the immunoprecipitatees in the presence of [γ-^32^P] ATP (250 μM) and assay buffer. At the designated time points, aliquots of the reaction mixture were taken out and subsequently analysed by SDS-PAGE followed by autoradiography. The reaction mixtures containing Src (K295M) only were the negative controls. Autoradiogram images were analyzed using ImageJ to obtain the relative density value of each band

To establish the biological relevance of the non-catalytic inhibitory mechanism of Chk we discovered in our in vitro studies, we conducted experiments with colorectal cancer cells to determine if Chk can employ this inhibitory mechanism to suppress SFK activity in cells. We previously demonstrated that Csk but not Chk is expressed in some colorectal cancer cell lines and in colon cancer biopsies [18]. Since SFKs are over-activated in both the early and late stages of colorectal cancer development [70–73], we aim to investigate how differential expression of Csk and Chk influences the activity of Src. Extensive screening of a wide spectrum of colorectal cancer cell lines revealed that Chk expression is suppressed while Csk is still expressed in these cell lines (manuscript in preparation). We chose one of these cell lines, DLD1, as a representative example for further biochemical analysis. We determined the specific enzymatic activity of Src immunoprecipitated from DLD-1 cells. For comparison, we also determined the specific enzymatic activity of active recombinant Src, which had not been activated by autophosphorylation prior to the in vitro assays. Figure 6a shows that Src isolated from DLD1 cells exhibited a significantly higher specific enzymatic activity than that of the recombinant unphosphorylated Src, indicating that Src is over-activated in DLD1 cells. When the mRNA levels of Csk and Chk were compared, we found that the Csk mRNA level is ~700-fold higher than that of Chk (Fig. 6b). Furthermore, we failed to detect Chk expression with two distinct Chk antibodies we generated and two Chk antibodies we purchased from commercial sources (data not shown). These results suggest that Chk expression is suppressed in DLD1 cells.

To investigate why Src is over-activated even in the presence of Csk, we immunoprecipitated Csk from the DLD1 cell lysate to eliminate the possibility that the endogenous Csk is inactive. We found that Csk could phosphorylate the kinase-dead Src (K295M) mutant (Fig. 6c). Our results suggest that while the Csk expressed in DLD1 cells is catalytically active, it cannot constrain Src kinase activity in these cells.

**Fig. 7:**
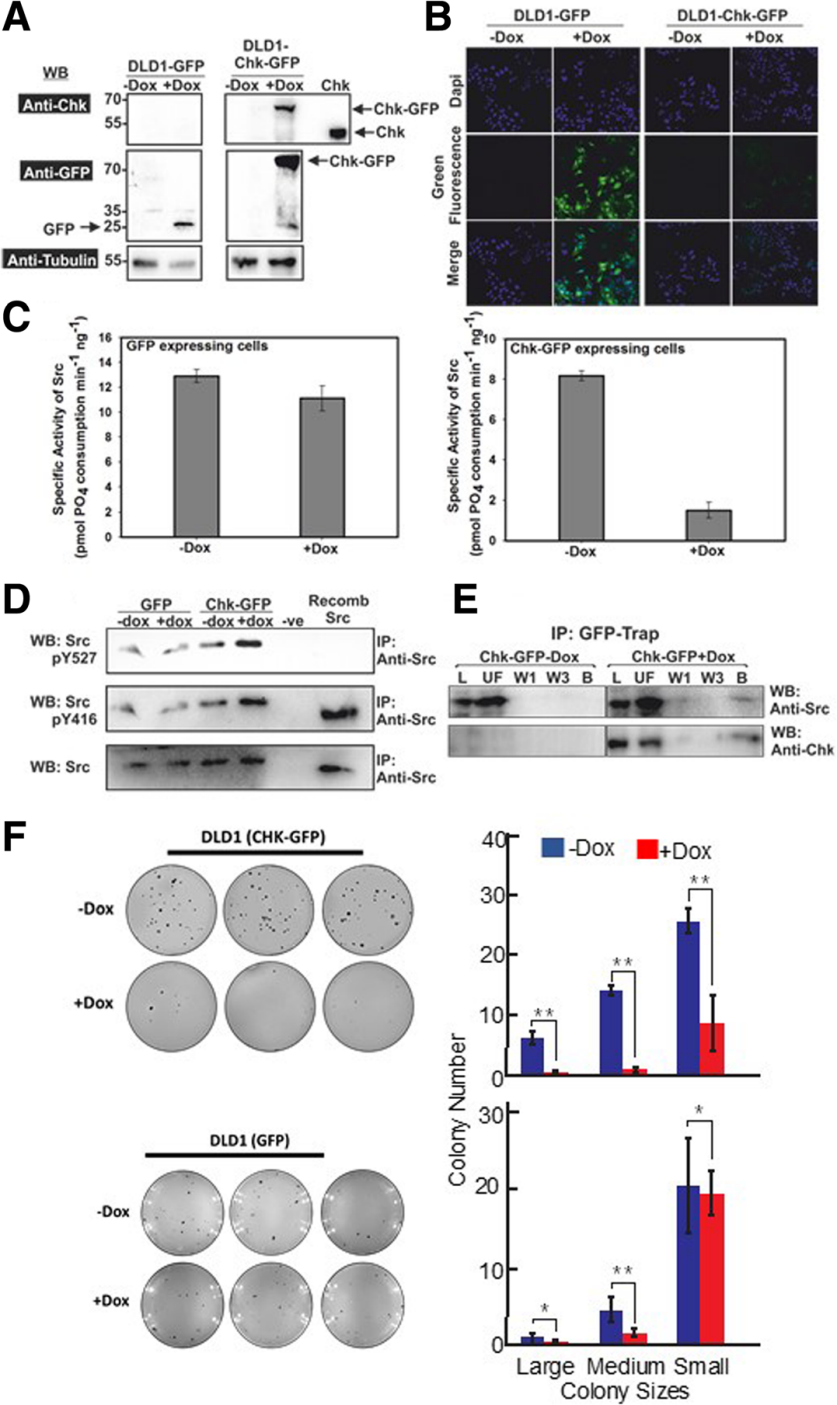
Recombinant Chk-GFP inhibits Src and forms complex with it in DLD1 cells. DLD1 cells were transduced with lentiviral vectors that directed expression of GFP or Chk-GFP under the control of 5 mg/ml doxycycline (dox). The transduced cells, referred to as DLD1-GFP or DLD1-Chk-GFP were examined for Src activity and formation of protein complexes containing Chk-GFP and Src. **a** Western Blot of cell lysates from the transduced cells in the presence and absence of Dox. Lysates (15–20 μg) were probed with anti-Chk and anti-GFP antibodies. Tubulin was used as the loading control (anti-tubulin) and the recombinant 52 kDa isoform of Chk was loaded as the positive control. **b** Immunofluoresence analysis of the transduced DLD1 cells before and after induced expression of GFP or Chk-GFP. The nuclei were stained with DAPI. **c** Src was immunoprecipitated from the transduced cell lines in the presence or absence of Dox, using anti-Src antibody. Its specific enzymatic activity in the immunoprecipitates was determined using Src-optimal peptide as the substrate. **d** Western blot of immunoprecipitated Src which was used to monitor its enzymatic activity. Anti-pY527, anti-pY416 and anti-Src antibodies were used to detect phosphorylation and presence of Src. Recombinant Src (truncated) was used as a positive control. **e** Co-immunoprecipitation of Src and Chk-GFP. GFP trap magnetic beads (Chromotek) were used to immunoprecipitate Chk-GFP from the transduced +/− dox cell lysates. Western blotting with anti-Src antibody was conducted to detect Src and Chk-GFP in the immunoprecipitated, the unbound fraction and the wash fractions. L = Original lysate, UF = Unbound fraction, W1 = Wash 1, W 3 = Wash 3, B = Proteins bound to the beads eluted with SDS sample buffer. **f** Soft agar colony-formation assay of GFP-expressing and Chk-GFP-expressing DLD1 cells. Expression of GFP and Chk-GFP was induced by doxycycline for 48 h prior to the assay. Experiment was performed in triplicates (*n* = 3 wells). Colonies were stained by 0.005% crystal violet for ~1 h. Images were taken using Biorad’s image lab software. *Left panels* colonies formed on soft agar by the transduced DLD1 cells. *Right panels* statistical analysis of colony-formation result. ImageJ software was used to count and define the sizes of colonies (Large: ≥ 20 pixels; Medium: 11–20 pixels; Small: 1–10 pixels). The colony number of each sample was demonstrated in the *left panel*, while the colony size was demonstrated in the *right panel*. The values represent the mean ± S.D. (*n* = 3 wells). *p > 0.05, **p < 0.05

To further explore the mechanism of Src over-activation in colorectal cancer cells, we expressed recombinant Chk-GFP in DLD1 cells (DLD1/Chk-GFP cells) under the control of a doxycycline-inducible promoter. DLD1 cells expressing GFP under the control of doxycycline (DLD1/GFP) were also generated as the negative control (Fig. 7a and b; Additional file 13: Figure S13). Src was immunoprecipitated from the lysates of both transduced cell lines for enzymatic analysis. Figure 7c shows that induced expression of GFP had no effect on Src activity while induced expression of Chk-GFP significantly inhibited Src kinase activity. Intriguingly, Chk-GFP did not cause any significant change in Src phosphoTyr-527 level, suggesting that Chk-GFP inhibited Src by the non-catalytic inhibitory mechanism, which is independent of tail phosphorylation (Fig. 7d). Indeed, the Western blot and proteomics data shown in Fig. 7e and Additional file 14: Table S2 show that Src co-immunoprecipitated with Chk-GFP but not with GFP from the lysates of the transduced DLD1 cells, indicating that Chk-GFP forms protein complexes with Src in the transduced DLD1 cells. Additionally, we also found that induced expression of Chk-GFP but not GFP suppressed anchorage-independent growth of the transduced DLD1 cells on soft agar (Fig. 7f). Taken together, our results suggest that recombinant Chk-GFP employs the non-catalytic mechanism to inhibit Src in the transduced DLD1 cells. Since Csk lacks the ability to efficiently inhibit Src with this non-catalytic inhibitory mechanism, its co-expression in DLD1 cells cannot suppress the kinase activity and oncogenic activity of Src.

## Conclusions

SFKs can adopt multiple active conformations in solution with their SH3, SH2 and kinase domains organised in different states of assembly [74]. These conformations differ in the accessibilities of the SH2, SH3 and kinase domains as well as their catalytic activities. For each active conformation, the accessibilities of the various functional domains govern its binding to a specific subset of cellular proteins, while the active site of the kinase domain selects the tyrosine residues in some of the bound proteins for phosphorylation. By adopting multiple active conformations, SFKs can bind and phosphorylate multiple subsets of cellular proteins, which control a wide array of cellular processes such as cell proliferation and neuronal survival [75].

Since aberrant activation of SFKs contributes to the onset and progression of many types of cancers, SFK activity in normal cells is repressed at most times by Csk and Chk. Although phosphorylation of the C-terminal tail tyrosine is a prerequisite for SFKs to adopt the closed inactive conformation, just its phosphorylation is insufficient to inhibit the multiple active conformations of SFKs. For example, C-terminal tail phosphorylation cannot overcome the activity of Src-family kinases in which their kinase domains are locked in the active conformation via activation loop phosphorylation. Thus, in addition to phosphorylation of the C-terminal tail tyrosine, additional inhibitory mechanisms capable of suppressing the activity of SFKs adopting multiple active conformations are needed. The non-catalytic inhibitory mechanism of Chk is one of these inhibitory mechanisms, which operates in conjunction with phosphorylation of the C-terminal tail tyrosine to constrain SFK activity in normal cells. Results of our biochemical studies to compare the efficiencies of Csk and Chk in phosphorylation and inhibition of SFKs by the non-catalytic inhibitory mechanism revealed the following findings. First, Csk phosphorylates the C-terminal tail tyrosine of Src much more efficiently than Chk. Second, Chk but not Csk can bind tightly to a constitutively activated SFK mutant. This ability of Chk allows it to effectively employ the non-catalytic inhibitory mechanism to inhibit the activated SFK mutant. Third, determinants governing the ability of Chk to tightly bind and inhibit the active SFK mutant reside in the kinase domain of Chk. We identified Arg-382 and Lys-387 in the αF-αG loop as two of these determinants. Besides these findings, our studies raise several issues related to the molecular basis of inhibition of SFKs by Csk and Chk and the identities of the determinants in Chk governing its tight binding to SFKs.

## Discussion

### What are the determinants in Chk governing its tight binding to SFKs?

Previously Levinson et al. reported that single point mutation of just one of the five conserved basic residues in the αD-helix and αF-αG loop of Csk significantly reduces its binding to Src [66]. In contrast, mutation of any one of the three arginine residues in the αD-helix (i.e. Arg-276, Arg-278 and Arg-280) of Chk has no impact on its binding to Hck (2PA-YEEI), while point mutation of either Arg-382 or Lys-387 led to a ~ 3-fold reduction in the rate of association (*k*_ON_) but no significant change in the rate of dissociation (*k*_OFF_) of Chk binding to Hck (2PA-YEEI) (Fig. 5g). There are two possible explanations for the differences in the impact of point mutations of the five conserved basic residues on binding of Csk and Chk to SFKs.

The first explanation is simple – besides the five conserved basic residues in the αD helix and αF-αG loop, the Chk kinase domain contains an additional motif mediating its tight binding to SFKs. Upon inspection of the highly homologous Csk and Chk kinase domain sequences (Additional file 15: Figure S12), we are unable to predict where this putative high affinity SFK-binding motif resides. It is possible that this motif is assembled only when the kinase domain adopts a unique conformation in solution. Determination of the three-dimensional structure of Chk in complex with a Src-family kinase domain may benefit the identification of this putative motif.

The second explanation relates to the ability to Csk and Chk to adopt the conformation that optimally presents the five conserved basic residues to SFKs in solution. For this explanation, we hypothesize that optimal alignment of the five conserved basic residues is sufficient for tight binding of Chk and Csk to SFKs. The structure of the Csk kinase domain in the Csk/Src complex determined by Levinson, et al. [66] represents the configuration in which these five conserved basic residues are optimally aligned for tight binding to SFKs (Additional files 11 and 15: Figures S10 and S12). We propose that the Chk kinase domain exhibits a much higher propensity than Csk kinase domain to adopt this configuration. Consequently, Chk is able to bind SFKs with an affinity much higher than that of Csk. Furthermore, as all five basic residues are optimally aligned for binding of SFKs, point mutation of just one of the five residues would have little negative impact on the affinity for Chk for SFKs. In contrast, the kinase domain of Csk in solution prefers to adopt the configuration in which only a few of the five conserved basic residues are optimally aligned for binding to SFKs. Thus, point mutation of any one of the five basic residues imposes a much more severe impact on its affinity to SFKs [66]. In addition to explaining the differences in impact of the point mutations of the conserved basic residues on binding of Csk and Chk to SFKs, this interpretation also explains why Chk is capable of binding SFKs with an affinity much higher than that of Csk.

In support of the second explanation, we previously demonstrated that the kinase-inactivating K221 M mutation completely abolished the ability of Chk to bind and inhibit SFKs [13]. Lys-221 corresponds to the conserved β3 strand lysine, which electrostatically interacts with the conserved glutamate residue (Glu-235 of Chk) of the αC helix in protein kinases adopting the active conformation. Our previous findings suggest that the Lys-221/Glu-235 interactions are needed for Chk kinase domain to adopt the active conformation of which the five conserved basic residues are optimally aligned for tight binding of SFKs. Future studies to define the structures of Chk/Src complex will reveal which of these explanations is correct.

### How does Chk inhibit SFKs in cells?

Comparison of the activity of Src in tumour and normal tissues revealed that Src activity in mammary and colorectal cancer tissues is 4- to 20-fold higher than that in normal tissues. Given that their autophosphorylation can lead to a ~ 20-fold increase in activity, it is logical to assume that only a relatively small portion of the total Src molecules expressed in cancer cells needs to be fully activated by autophosphorylation to induce the oncogenic phenotypes. Presumably, as long as Chk is expressed at a level that is sufficient to tightly bind and inhibit this small portion of active Src, it can function as a tumour suppressor to inhibit the growth and survival of cancer cells. Since active Src is targeted to the plasma membrane upon autophosphorylation at Tyr-416, Chk also needs to colocalize with these plasma membrane-bound activated Src molecules to effectively bind to them and inhibit their activity.

The kinetic parameters of Chk binding and phosphorylation of SFKs presented in this manuscript were determined in the in vitro conditions in the absence of other regulatory proteins of Chk and SFKs. Thus, our findings only reflect a few aspects of the multi-step process of inhibition of SFKs by Chk in cells. Similar to Csk, Chk resides predominantly in cytosol and nucleus (Fig. 5b, Additional file 13: Figure S13) [76]. Chk therefore needs to bind to a membrane-bound scaffolding protein for its recruitment to the plasma membrane, where it can efficiently inhibit SFKs by both the non-catalytic inhibitory mechanism and phosphorylation of their C-terminal tail tyrosine. The binding of Chk to this unknown scaffolding protein may also modulate its affinity for SFKs. Future investigation should focus on identifying this putative Chk-binding protein scaffold and defining how Chk binding to this putative protein scaffold affects its affinity for SFKs and the efficiency of its inhibition of SFKs. Figure 5 shows that induced expression of recombinant Chk-GFP led to a significantly reduction (>80%) of Src activity in the transduced DLD1 cells. Thus, the Chk-GFP expressing DLD1 cell line developed by us can be a useful tool to identify this putative Chk-binding scaffolding protein.

### Is Chk a tumour suppressor downregulated in colorectal cancer cells?

It is well documented that over-activation of SFKs directs colorectal cancer tumorigenesis, disease progression and development of drug resistance [2, 70, 77–79]. Cordero et al. reported that Src, which is over-activated following loss of expression of adenomatous polyposis coli (Apc), is required for colon cancer tumorigenesis [2]. Results of our studies of the DLD1 cells suggest that loss of the non-catalytic inhibitory mechanism of Chk resulting from suppression of its expression, contributes to Src over-activation and in turn to colorectal cancer tumorigenesis and progression. Future investigation may focus on understanding if and how Apc loss suppresses Chk expression. Of relevance, the promoter of *Chk* gene is hypermethylated in tumour biopsies collected from colorectal cancer patients [80, 81]. Hypermethylation of promoters is an epigenetic mechanism often responsible for silencing tumor suppressor gene expression. Methylation of specific CpG islands in the *Chk* gene promoter therefore represents one plausible mechanism of Chk expression in tumors.

## Additional files

Additional file 1: Figure S1. Hypothetical conformations of the SFK and mutants used in this study. The two SFK members Src and Hck were chosen for our analyses. The N-terminal unique domain of both Src and Hck mutants is replaced by poly-His (His_6_) and Flag tags. For Src (K295M), Lys-295 critical to binding ATP is replaced by Met, hence the mutant is inactive. For Hck (2PA-YEEI), two conserved prolines in the SH2-kinase linker are replaced by alanines (referred to as the 2PA mutation), and the C-terminal YQQQP motif is replaced by the YEEIP motif (referred to as the YEEI mutation). The 2PA mutation prevents the Hck mutant from adopting the “closed” inactive conformation because the two conserved prolines are critical for intramolecular interactions between the PQKP motif with the SH3 domain. The mutant is therefore constitutively active. The YEEI mutation converts the motif around the C-terminal tail tyrosine into YEEIP motif which is an optimal phosphorylation sequence of SFKs. The constitutively active mutant undergoes autophosphorylation at both the conserved autophosphorylation site (Y_A_) and the C-terminal tail tyrosine (Y_T_). Upon phosphorylation, the pYEEIP motif can bind to the SH2 domain of the mutant with high affinity. Based upon the results of the structural and biochemical analyses of the Src and Hck mutants presented by Lerner, et al. [40] and Cowan-Jacob, et al. [82], the predicted conformations of the Src and Hck mutants are depicted in the left column. (TIFF 623 kb)

Additional file 2: **Figure S2**. Coomassie blue-stained gels showing the purity of the recombinant Csk, Chk, Src, Src (K295 M) mutant and Hck (2PA-YEEI) mutant used in this study. (TIFF 325 kb)

Additional file 3: **Figure S7**. Extracted ion chromatogram (XIC) for the C terminal peptide containing the Tyr-527 phosphorylation site of Src in the presence and absence of Csk. **A-D** Src (K295M) alone (A) Src (K295M) with Csk (B), Src alone (C) and Src with Csk (D) were incubated with ATP and assay buffer. Peptides are identified by Mascot in accordance of retention time. Retention time and mass error (ppm) are shown. **E.** Isotope dot product (idotp) comparing the observed and theoretical distribution of the precursor isotope with 1.0 being an optimal match. (TIFF 688 kb)

Additional file 4: Figure S3. Csk and Chk recognise identical substrate specificity determinants. **A**. Peptides in the arrayed combinatorial peptide library were designed with each peptide containing the general sequence G-A-X-X-X-X-X-Y-X-X-X-X-A-G-K-K (biotin). After incubation with Csk in the presence of [γ-^33^P] ATP, phosphorylation of peptides in the library was detected by autoradiography. The autoradiograms of both runs of peptide library screen are shown. The intensity of each spot signifies the extent of phosphorylation of peptides with the indicated residue at the specified position relative to the tyrosine residue at position zero. **B.** Identification of the substrate specificity determinants of Csk by positional scanning peptide library screening. Peptides in the arrayed combinatorial peptide library were designed with each peptide containing the general sequence G-A-X-X-X-X-X-Y-X-X-X-X-A-G-K-K (biotin). After incubation with Csk in the presence of [γ-^33^P] ATP, phosphorylation of peptides in the library was detected by autoradiography. The intensity of each spot signifies the extent of phosphorylation of peptides with the indicated residue at the specified position relative to the tyrosine residue at position zero. Both Csk and Chk were used to identify the substrate specificity determinants. **C.** Tables summarising the residues positively selected at the specified position by Csk (upper) and Chk (lower). The values of normalised quantified spot intensities are noted within the parentheses. The lower table is adapted from our previous published findings [52]. Only values greater or equal to 1.5 are shown. The strong signal for peptides with fixed tyrosine residues independent of position is probably due to an artefact as there are two phosphorylatable residues in the peptides. The Csk optimal phosphorylation sequence (x-D-E-x-(Ф/E/D)-Y-Ф-x-Ф-x) shown in panel C is very similar to the Csk-optimal phosphorylation sequence (EEEIYFFF) determined by Sondhi et al. [83] using the combinatorial peptide library approach. (TIFF 899 kb)

Additional file 5: Figure S4. Lineweaver-Burk plots of the rates of phosphorylation of the Csk/Chk-optimal peptide by Csk and Chk. For the phosphorylation reactions, 0.2 μM Chk-His6 or 0.2 μM Csk were used to phosphorylate the Csk/Chk optimal peptide substrates at 0–500 μM. The initial velocities were plotted against the substrate peptide concentrations. The data points were fitted into Michaelis-Menten equation and transformed to Lineweaver-Burk plots. This data set shown is a representative of three. The kinetic parameters demonstrated are the Michaelis-Menten constant (K_m_), the catalytic constant (*k*_*cat*_) and the specificity constant (*k*_*cat*_/K_m_). (TIFF 437 kb)

Additional file 6: Figure S5. Mass spectra and phosphopeptide mapping of tryptic fragments containing the phosphorylated Tyr-416 and Tyr-527 of Src. **A.** Mass spectra of the tryptic fragment containing phospho-Tyr-416 derived from autophosphorylated recombinant Src (upper panel) and the tryptic fragment containing phospho-Tyr-527 derived from recombinant Src (K295M) phosphorylated by Csk (lower panel). The phosphorylated proteins were generated by incubation of recombinant Src alone or incubation of Src (K295M) and Csk with 250 μM for 30 min at 30 °C **B.** Phosphopeptide mapping of (i) autophosphorylated Src, (ii) Src (K295M) phosphorylated by Csk and (iii) Src phosphorylated by Csk in the presence of [γ-^32^P] ATP (specific radioactivity: ~300 cpm/pmol) under the same conditions as described in panel A. The radioactively phosphorylated Src and Src (K295M) were subject to SDS-PAGE and electrotransferred to nitrocellulose membranes. The membrane strips containing the phosphorylated Src and Src (K295M) were incubated with trypsin. The resultant tryptic phosphopeptides were analysed by two-dimensional phosphopeptide mapping. The two arrows at the sample origin of the key show the directions of movement of tryptic phosphopeptides in electrophoresis (TLE, first dimension) and chromatography (TLC, second dimension). The key denotes the migration patterns of tryptic phosphopeptides derived from Tyr-416 and Tyr-527. O: origin. (TIFF 927 kb)

Additional file 7: Figure S6. Extracted ion chromatogram (XIC) of the phosphopeptide containing the autophosphorylated Tyr-416 of Src and Src (K295M) in the presence and absence of Csk. **A-D** Src (K295M) mutant alone (A), Src (K295M) with Csk (B), Src alone (C) and Src with Csk (D) were allowed to undergo phosphorylation in vitro. Peptides are identified by Mascot in accordance of their retention time. Retention time and mass error (ppm) are shown. **E.** Isotope dot product (idotp) comparing the observed and theoretical distribution of the precursor isotope with 1.0 being an optimal match. (TIFF 1102 kb)

Additional file 8: Table S1. Determination of the molecular binding activity of Chk, Csk and Csk-Chk Chimera to immobilized Hck(2PA-YEEI) by surface plasmon spectroscopy. (PDF 447 kb)

Additional file 9: Figure S8. Purified Csk-Chk chimera is intact, folded and catalytically active. **A.** Primary structure of the engineered Csk-Chk Chimera. **B.** SDS-PAGE of purified Csk-Chk Chimera shows Csk-Chk Chimera was purified to more than 95% purity. **C.** Circular Dichroism Spectrum of Csk-Chk Chimera demonstrates minima at 208 nm and 222 nm wavelengths, indicating presence of α-helices. **D.** Kinetic parameters of the phosphorylation of the Csk/Chk optimal peptide by Chk, Csk-Chk chimera and Csk. The kinetic parameters include Michaelis-Menten constant (K_M_), *k*_*cat*_ and *k*_*cat*_/K_M_. (TIFF 598 kb)

Additional file 10: Figure S9. Comparison of the molecular binding activities (M.B.A.) of Chk, Csk-Chk chimera and Csk. The M.B.A. values of Chk, Csk-Chk Chimera and Csk were plotted against at the designated concentrations used in the surface plasmon resonance spectroscopic analysis of the kinetics of their binding to the immobilised Hck (2PA-YEEI) (Figs. 4 and 5). Calculation of the M.B.A. values was presented in Additional file 8: Table S1. (TIFF 309 kb)

Additional file 11: Figure S10. Key residues involved in electrostatic and hydrophobic interactions at the Src-Csk interface and recombinant Chk mutants generated. A. The five conserved basic residues in the αD-helix and αF-αG loop of Csk interact electrostatically and hydrophobically with Lys-442 and acidic and hydrophobic residues in the αH-αI loop, αI and αI’ helices of Src. The image was generated by Molsoft L.L.C. using the coordinates of the structure of Csk/Src complex (PDB ID: 3D7T). The C-terminal tail tyrosine (Tyr-527) is shown in green. B. Alignment showing the five conserved basic residues in the Csk and Chk sequences. Lys-442 and the C-terminal tail sequence of Src at the Csk/Src interface are shown. The key residues participating in direct interactions with Csk are in red. C. Coomassie blue-stained gels showing the purity of purified Chk and its mutants used in this study. (TIFF 279 kb)

Additional file 12: Figure S11. Kinetic analysis of phosphorylation of Chk/Csk-optimal peptide by Chk (R276A), Chk (R278A), Chk (R280A), Chk (R382A) and Chk (K387A). **A-F.** Lineweaver-Burk plots of wild-type Chk (A), Chk (R276A) (B), Chk (R278A) (C), Chk (R280A) (D), Chk (R382A) (E) and Chk (K387A) (F). **G.** Kinetic parameters of the catalytic activity of Chk and its mutants including the Michaelis-Menten constant (K_M_), the catalytic constant (*k*_*cat*_) and the specificity constant (*k*_*cat*_/K_M_). (TIFF 463 kb)

Additional file 13: Figure S13. Immunofluorescence analysis of transduced DLD1 cells expressing the recombinant CHK-GFP. A close-up image of one of the “merge” panels in Fig. 7b. Green: CHK-GFP; blue: DAPI stain. (TIFF 302 kb)

Additional file 14: Table S2. Identification of tryptic fragments derived from Src co- immunoprecipitated with Chk-GFP from the DLD-1-Chk-GFP cell lysate. Proteins immunoprecipitated from lysate of the DLD-1-Chk-GFP cells and that of the DLD-1-GFP cells (Control) were processed and digested with trypsin as described previously by Ang and Nice [56]. The tryptic fragments were analysed by LC-MS/MS as described in the experimental procedures. The SRC peptides identified by LC MS/MS and using the SEQUEST search engine are listed. A total of 4 different peptides were identified with high confidence at 1% FDR. The identify of these peptides were further validated with the Percolator algorithm for discrimination between correct and incorrect spectrum identifications as described by Kall, et al [84]. These fragments were not detected in the anti-GFP immunoprecipitate of the DLD-1-GFP cell lysate (Control lysate), suggesting that Src was specifically bound to Chk-GFP in the DLD-1-Chk-GFP cells. (PDF 93 kb)

Additional file 15: Figure S12. Alignment of the sequences of Csk and Chk kinase domains. Arg-279, Arg-281 and Arg-283 of αD helix and the αD/αE loop, and Arg-384 and Arg-389 of αF/αG loop of Csk and the homologous basic residues in Chk are shown in red. (TIFF 564 kb)

## Abbreviations

Chk: Csk-homologous kinase. It is also referred to as megakaryocyte-associated tyrosine kinase (Matk)
Csk: C-terminal Src kinase
Hck: Hematopoietic Cell Kinase
p130^Cas^: Cadherins-associated Src substrate. It is also referred as catenin delta-1
