# Supplementary figures and images for: Csk-homologous kinase (Chk) is an efficient inhibitor of Src-family kinases but a poor catalyst of phosphorylation of their C-terminal regulatory tyrosine
Source: Cell Commun Signal. 2017 Aug 7;15:29. doi: 10.1186/s12964-017-0186-x (PMC5547543; doi:10.1186/s12964-017-0186-x)

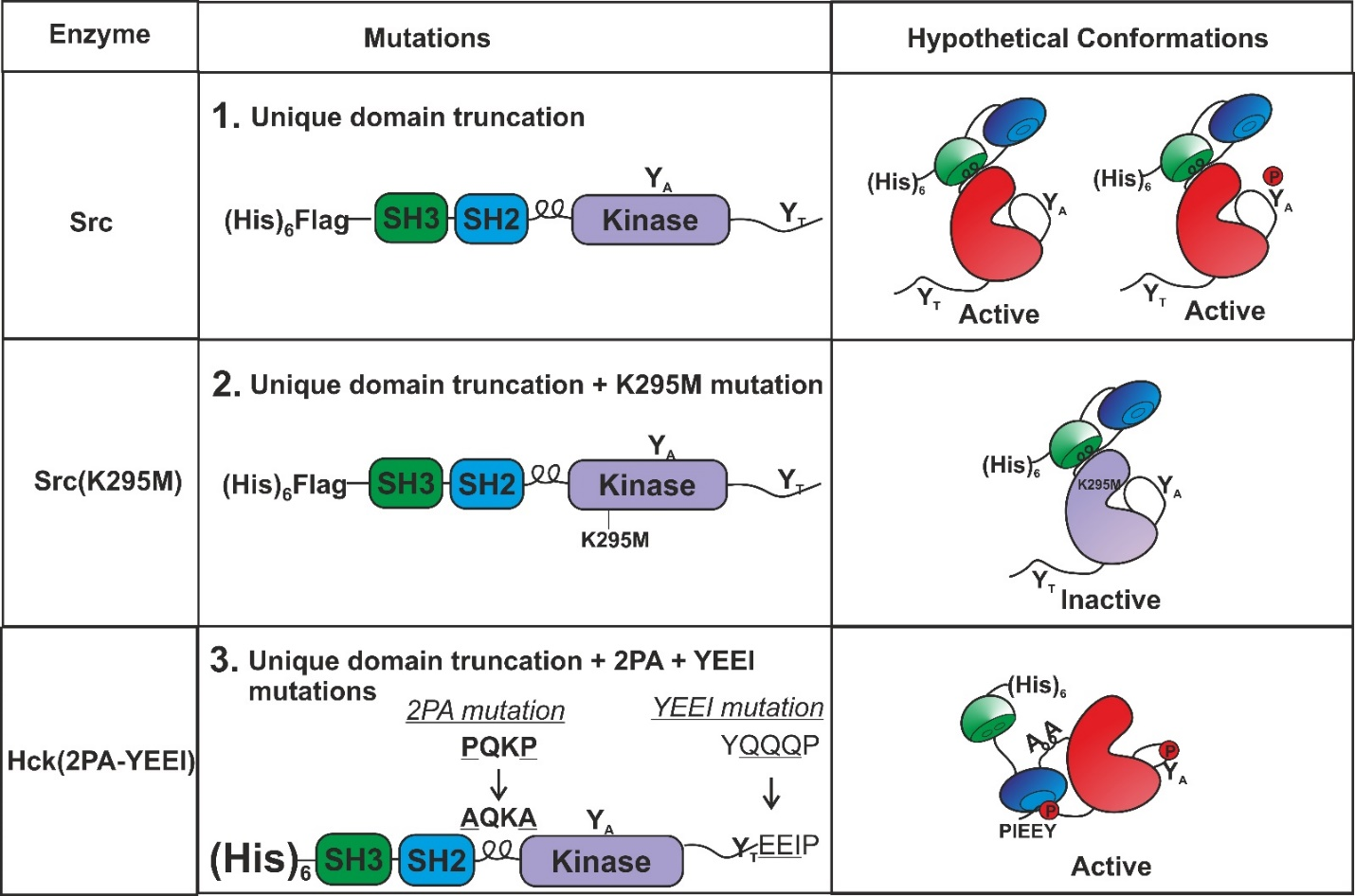

Supplement: Supplementary file 1 — Hypothetical conformations of the SFK and mutants used in this study. The two SFK members Src and Hck were chosen for our analyses. The N-terminal unique domain of both Src and Hck mutants is replaced by poly-His (His6) and Flag tags. For Src (K295M), Lys-295 critical to binding ATP is replaced by Met, hence the mutant is inactive. For Hck (2PA-YEEI), two conserved prolines in the SH2-kinase linker are replaced by alanines (referred to as the 2PA mutation), and the C-terminal YQQQP motif is replaced by the YEEIP motif (referred to as the YEEI mutation). The 2PA mutation prevents the Hck mutant from adopting the “closed” inactive conformation because the two conserved prolines are critical for intramolecular interactions between the PQKP motif with the SH3 domain. The mutant is therefore constitutively active. The YEEI mutation converts the motif around the C-terminal tail tyrosine into YEEIP motif which is an optimal phosphorylation sequence of SFKs. The constitutively active mutant undergoes autophosphorylation at both the conserved autophosphorylation site (YA) and the C-terminal tail tyrosine (YT). Upon phosphorylation, the pYEEIP motif can bind to the SH2 domain of the mutant with high affinity. Based upon the results of the structural and biochemical analyses of the Src and Hck mutants presented by Lerner, et al. [40] and Cowan-Jacob, et al. [82], the predicted conformations of the Src and Hck mutants are depicted in the left column. (TIFF 623 kb) [file 12964_2017_186_MOESM1_ESM.tif]

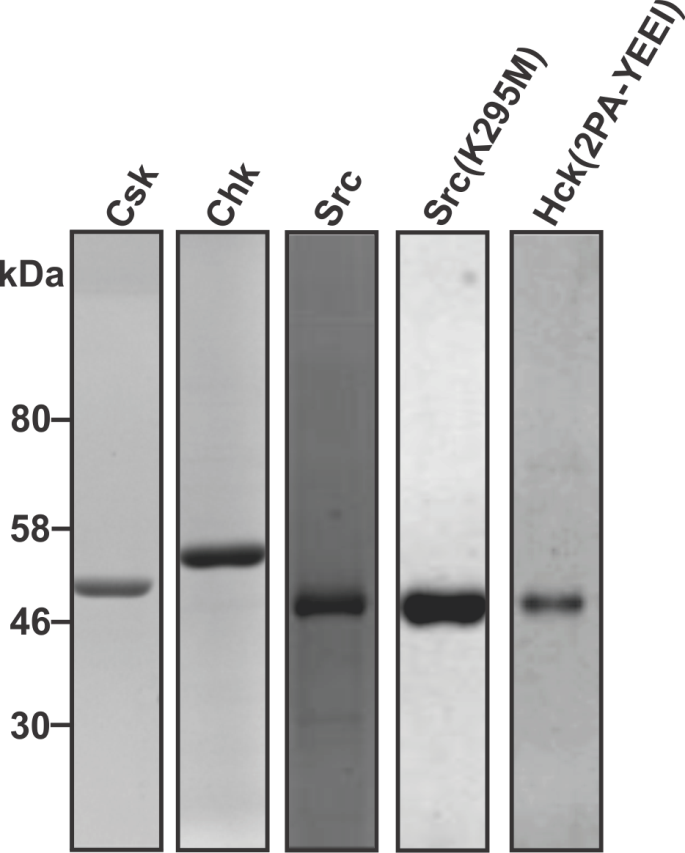

Supplement: Supplementary file 2 — Figure S2. Coomassie blue-stained gels showing the purity of the recombinant Csk, Chk, Src, Src (K295 M) mutant and Hck (2PA-YEEI) mutant used in this study. (TIFF 325 kb) [file 12964_2017_186_MOESM2_ESM.tif]

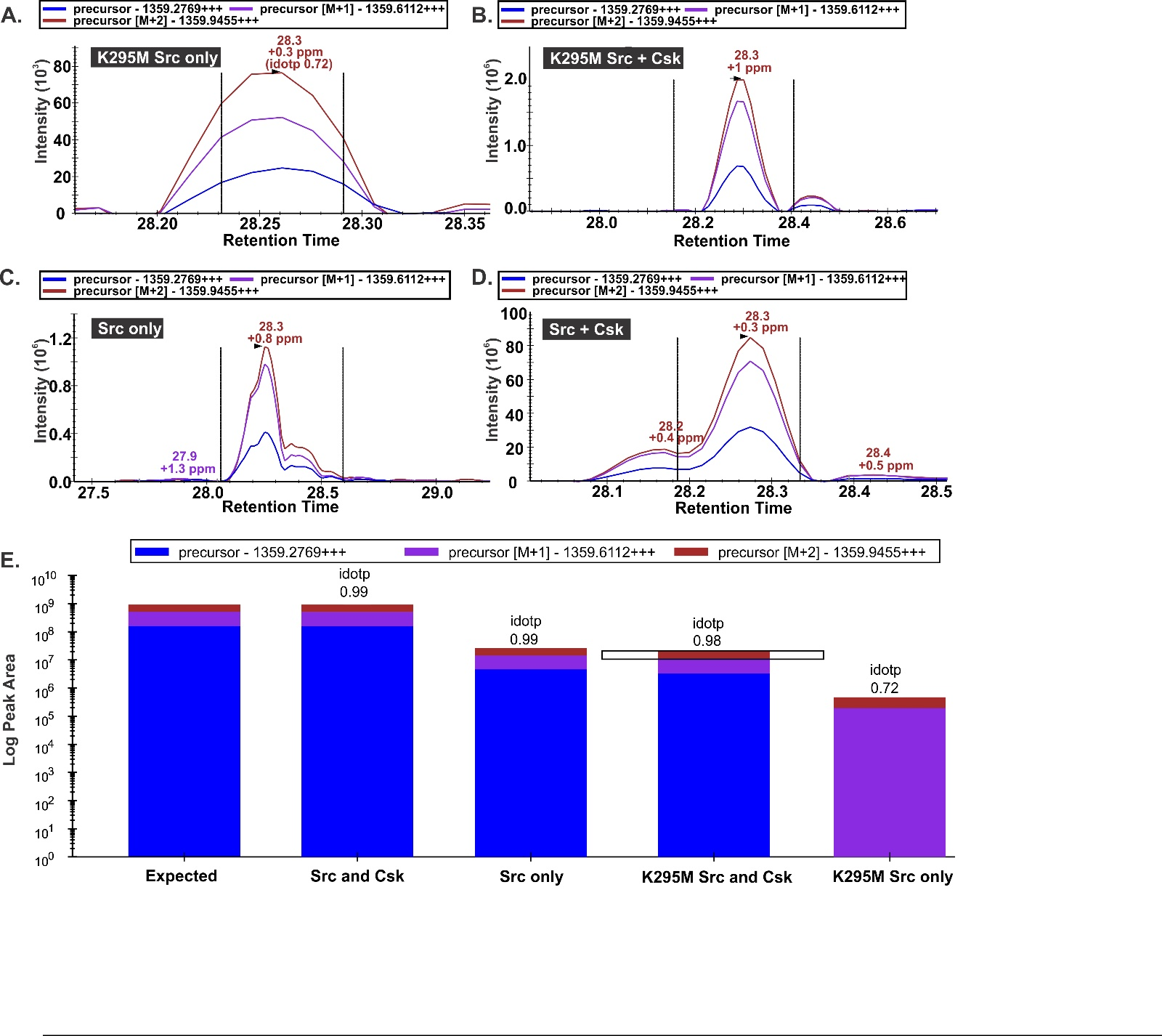

Supplement: Supplementary file 3 — Figure S7. Extracted ion chromatogram (XIC) for the C terminal peptide containing the Tyr-527 phosphorylation site of Src in the presence and absence of Csk. A-D Src (K295M) alone (A) Src (K295M) with Csk (B), Src alone (C) and Src with Csk (D) were incubated with ATP and assay buffer. Peptides are identified by Mascot in accordance of retention time. Retention time and mass error (ppm) are shown. E. Isotope dot product (idotp) comparing the observed and theoretical distribution of the precursor isotope with 1.0 being an optimal match. (TIFF 688 kb) [file 12964_2017_186_MOESM3_ESM.tif]

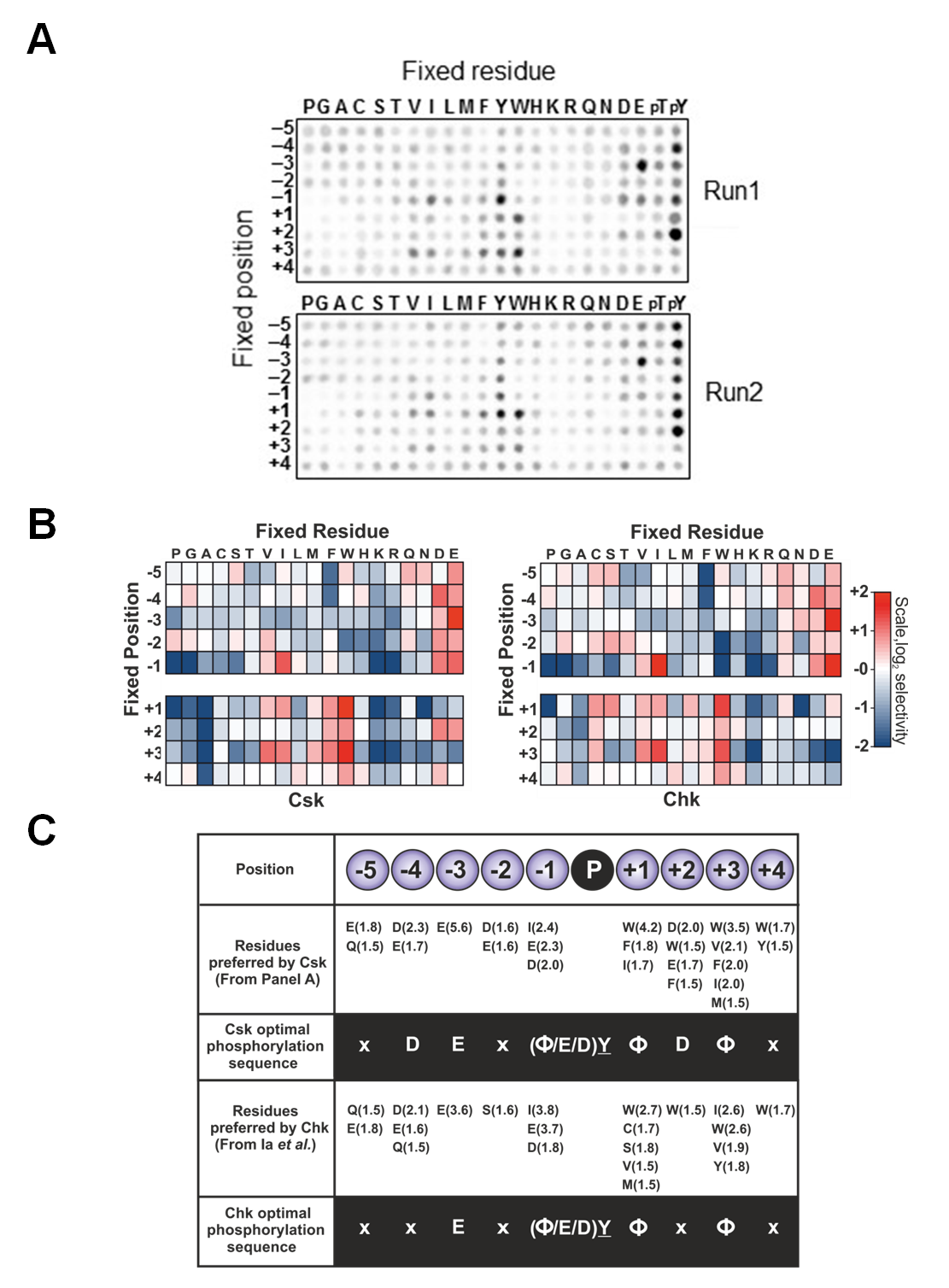

Supplement: Supplementary file 4 — Csk and Chk recognise identical substrate specificity determinants. A. Peptides in the arrayed combinatorial peptide library were designed with each peptide containing the general sequence G-A-X-X-X-X-X-Y-X-X-X-X-A-G-K-K (biotin). After incubation with Csk in the presence of [γ-33P] ATP, phosphorylation of peptides in the library was detected by autoradiography. The autoradiograms of both runs of peptide library screen are shown. The intensity of each spot signifies the extent of phosphorylation of peptides with the indicated residue at the specified position relative to the tyrosine residue at position zero. B. Identification of the substrate specificity determinants of Csk by positional scanning peptide library screening. Peptides in the arrayed combinatorial peptide library were designed with each peptide containing the general sequence G-A-X-X-X-X-X-Y-X-X-X-X-A-G-K-K (biotin). After incubation with Csk in the presence of [γ-33P] ATP, phosphorylation of peptides in the library was detected by autoradiography. The intensity of each spot signifies the extent of phosphorylation of peptides with the indicated residue at the specified position relative to the tyrosine residue at position zero. Both Csk and Chk were used to identify the substrate specificity determinants. C. Tables summarising the residues positively selected at the specified position by Csk (upper) and Chk (lower). The values of normalised quantified spot intensities are noted within the parentheses. The lower table is adapted from our previous published findings [52]. Only values greater or equal to 1.5 are shown. The strong signal for peptides with fixed tyrosine residues independent of position is probably due to an artefact as there are two phosphorylatable residues in the peptides. The Csk optimal phosphorylation sequence (x-D-E-x-(Ф/E/D)-Y-Ф-x-Ф-x) shown in panel C is very similar to the Csk-optimal phosphorylation sequence (EEEIYFFF) determined by Sondhi et al. [83] using the combinatorial pept [file 12964_2017_186_MOESM4_ESM.tif]

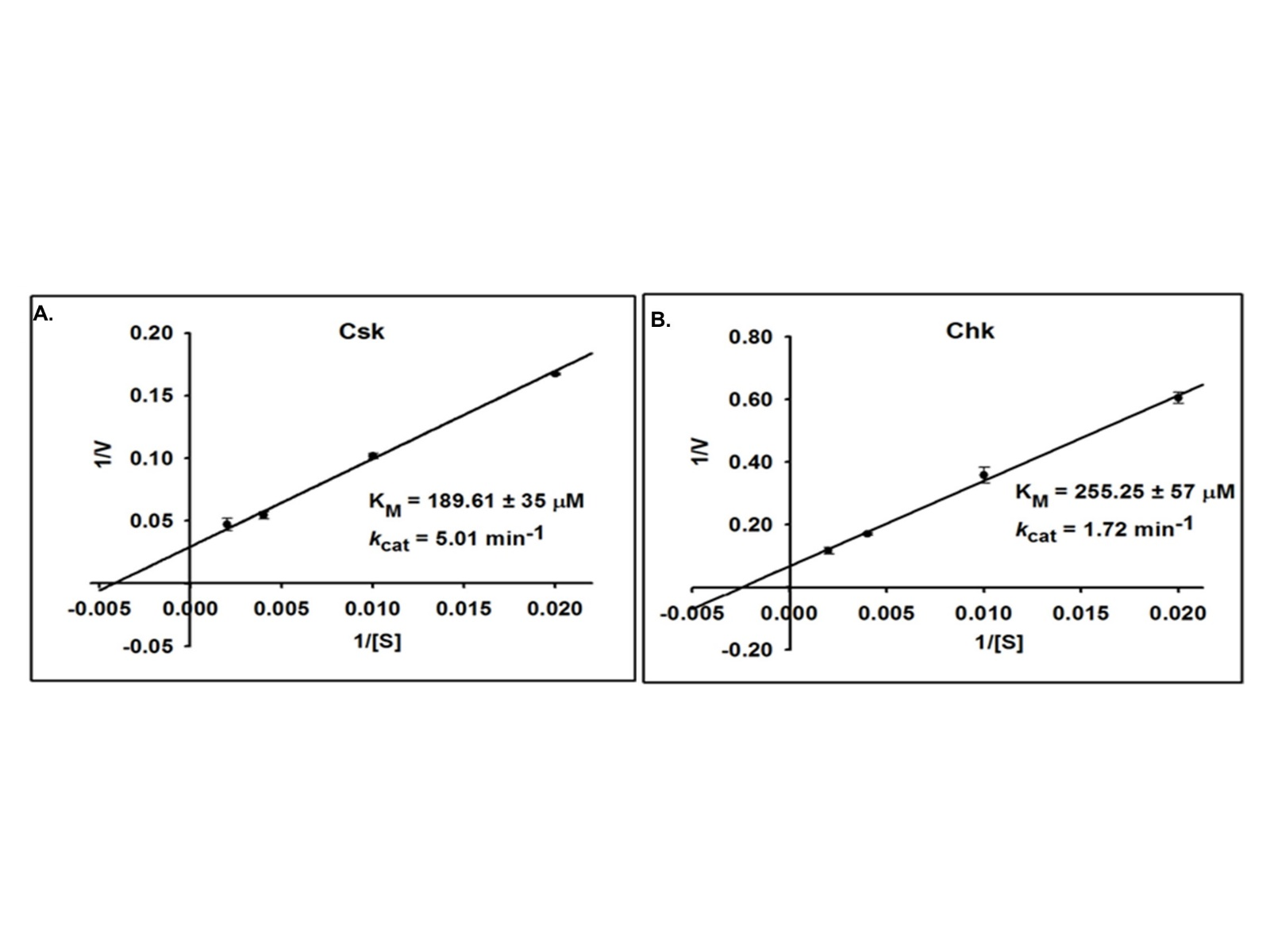

Supplement: Supplementary file 5 — Lineweaver-Burk plots of the rates of phosphorylation of the Csk/Chk-optimal peptide by Csk and Chk. For the phosphorylation reactions, 0.2 μM Chk-His6 or 0.2 μM Csk were used to phosphorylate the Csk/Chk optimal peptide substrates at 0–500 μM. The initial velocities were plotted against the substrate peptide concentrations. The data points were fitted into Michaelis-Menten equation and transformed to Lineweaver-Burk plots. This data set shown is a representative of three. The kinetic parameters demonstrated are the Michaelis-Menten constant (Km), the catalytic constant (k cat) and the specificity constant (k cat/Km). (TIFF 437 kb) [file 12964_2017_186_MOESM5_ESM.tif]

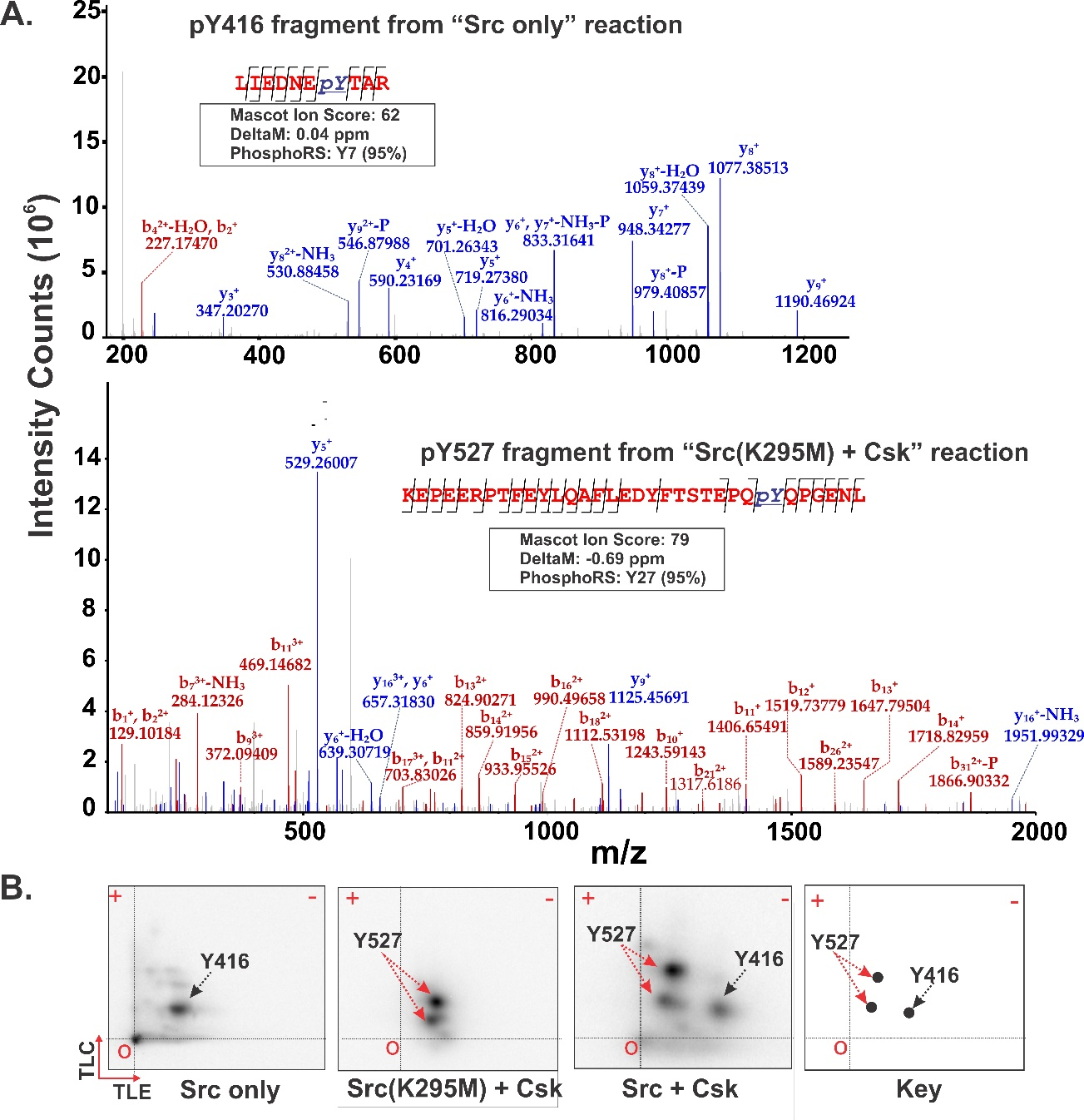

Supplement: Supplementary file 6 — Mass spectra and phosphopeptide mapping of tryptic fragments containing the phosphorylated Tyr-416 and Tyr-527 of Src. A. Mass spectra of the tryptic fragment containing phospho-Tyr-416 derived from autophosphorylated recombinant Src (upper panel) and the tryptic fragment containing phospho-Tyr-527 derived from recombinant Src (K295M) phosphorylated by Csk (lower panel). The phosphorylated proteins were generated by incubation of recombinant Src alone or incubation of Src (K295M) and Csk with 250 μM for 30 min at 30 °C B. Phosphopeptide mapping of (i) autophosphorylated Src, (ii) Src (K295M) phosphorylated by Csk and (iii) Src phosphorylated by Csk in the presence of [γ-32P] ATP (specific radioactivity: ~300 cpm/pmol) under the same conditions as described in panel A. The radioactively phosphorylated Src and Src (K295M) were subject to SDS-PAGE and electrotransferred to nitrocellulose membranes. The membrane strips containing the phosphorylated Src and Src (K295M) were incubated with trypsin. The resultant tryptic phosphopeptides were analysed by two-dimensional phosphopeptide mapping. The two arrows at the sample origin of the key show the directions of movement of tryptic phosphopeptides in electrophoresis (TLE, first dimension) and chromatography (TLC, second dimension). The key denotes the migration patterns of tryptic phosphopeptides derived from Tyr-416 and Tyr-527. O: origin. (TIFF 927 kb) [file 12964_2017_186_MOESM6_ESM.tif]

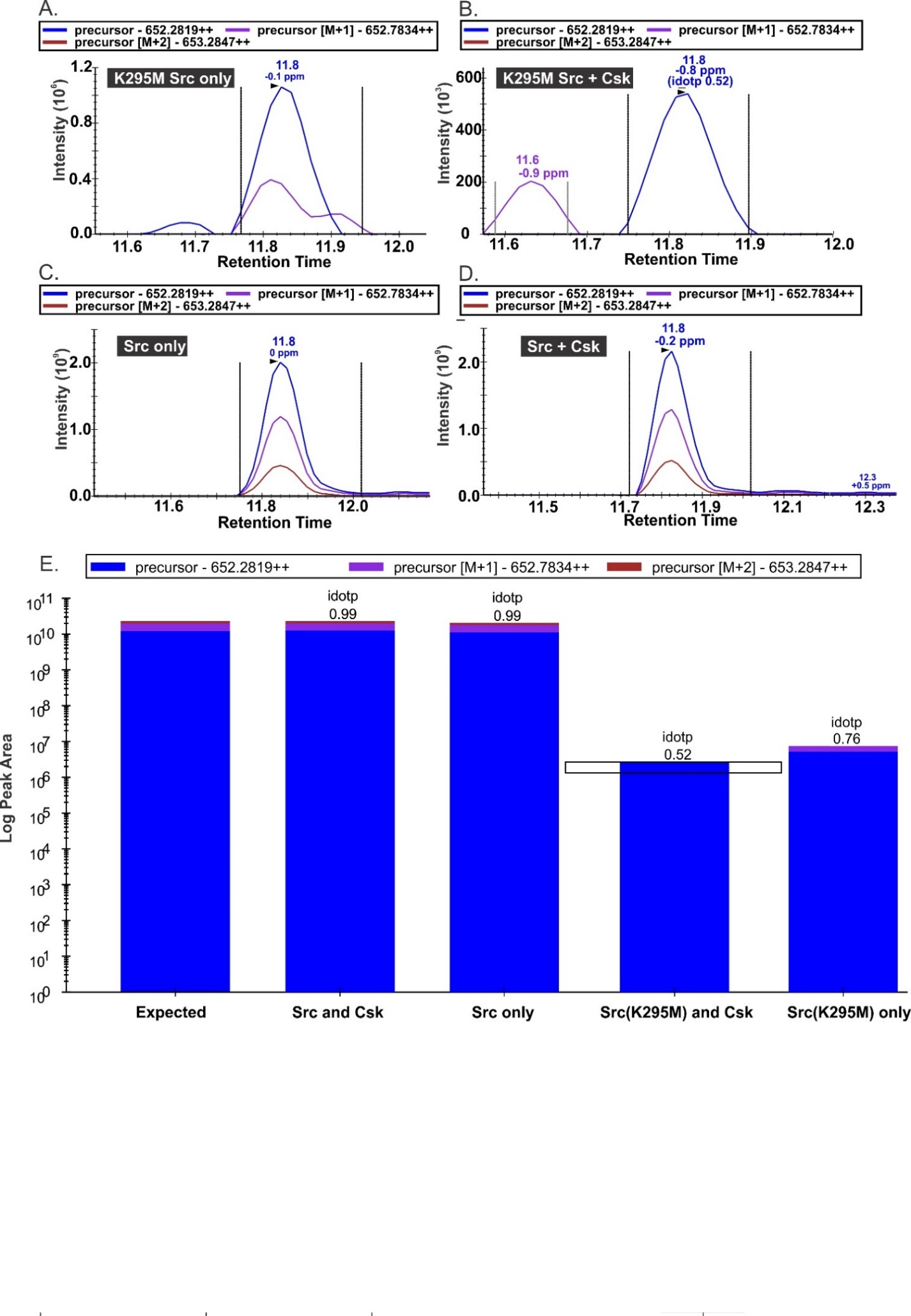

Supplement: Supplementary file 7 — Extracted ion chromatogram (XIC) of the phosphopeptide containing the autophosphorylated Tyr-416 of Src and Src (K295M) in the presence and absence of Csk. A-D Src (K295M) mutant alone (A), Src (K295M) with Csk (B), Src alone (C) and Src with Csk (D) were allowed to undergo phosphorylation in vitro. Peptides are identified by Mascot in accordance of their retention time. Retention time and mass error (ppm) are shown. E. Isotope dot product (idotp) comparing the observed and theoretical distribution of the precursor isotope with 1.0 being an optimal match. (TIFF 1102 kb) [file 12964_2017_186_MOESM7_ESM.tif]

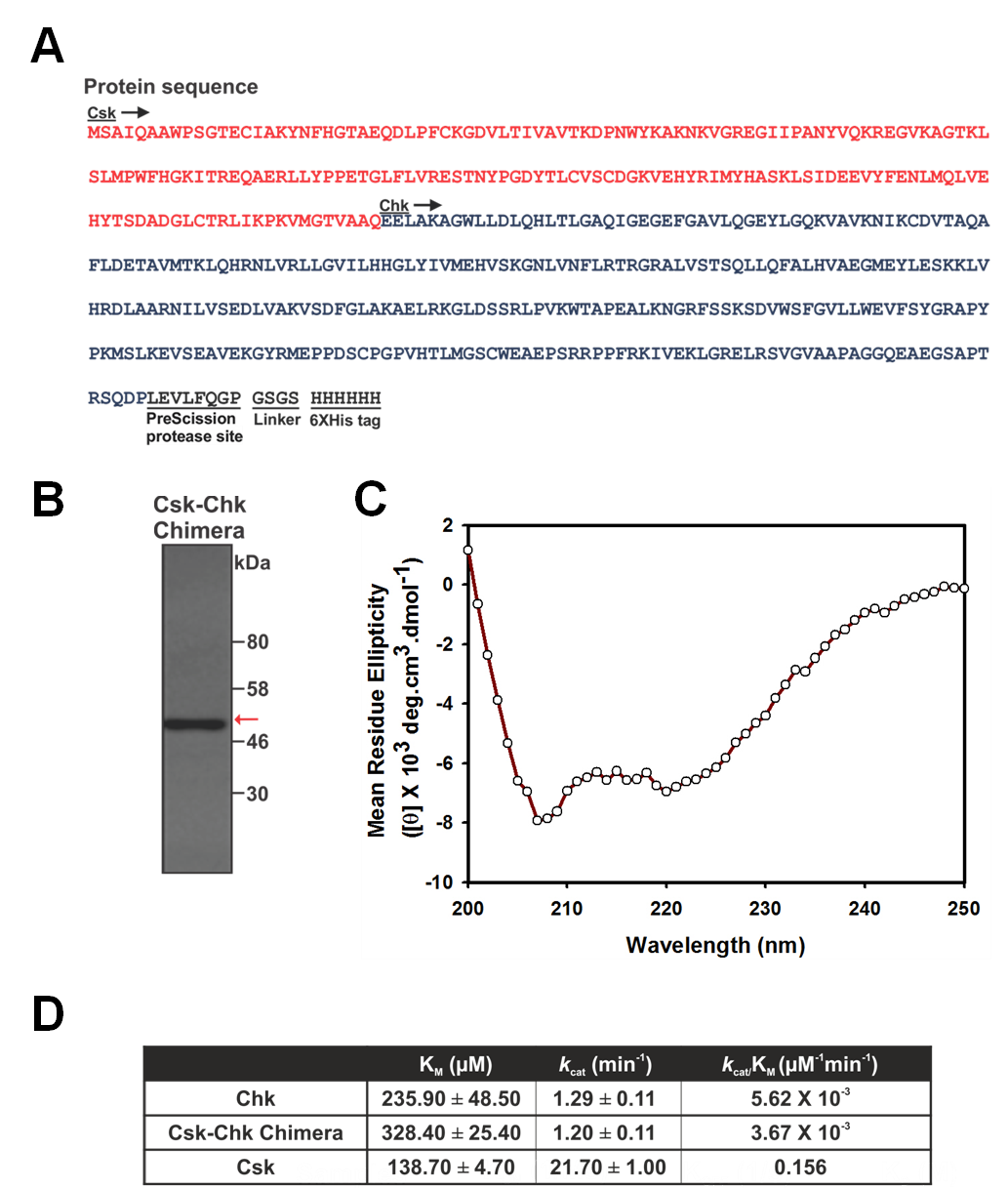

Supplement: Supplementary file 9 — Purified Csk-Chk chimera is intact, folded and catalytically active. A. Primary structure of the engineered Csk-Chk Chimera. B. SDS-PAGE of purified Csk-Chk Chimera shows Csk-Chk Chimera was purified to more than 95% purity. C. Circular Dichroism Spectrum of Csk-Chk Chimera demonstrates minima at 208 nm and 222 nm wavelengths, indicating presence of α-helices. D. Kinetic parameters of the phosphorylation of the Csk/Chk optimal peptide by Chk, Csk-Chk chimera and Csk. The kinetic parameters include Michaelis-Menten constant (KM), k cat and k cat/KM. (TIFF 598 kb) [file 12964_2017_186_MOESM9_ESM.tif]

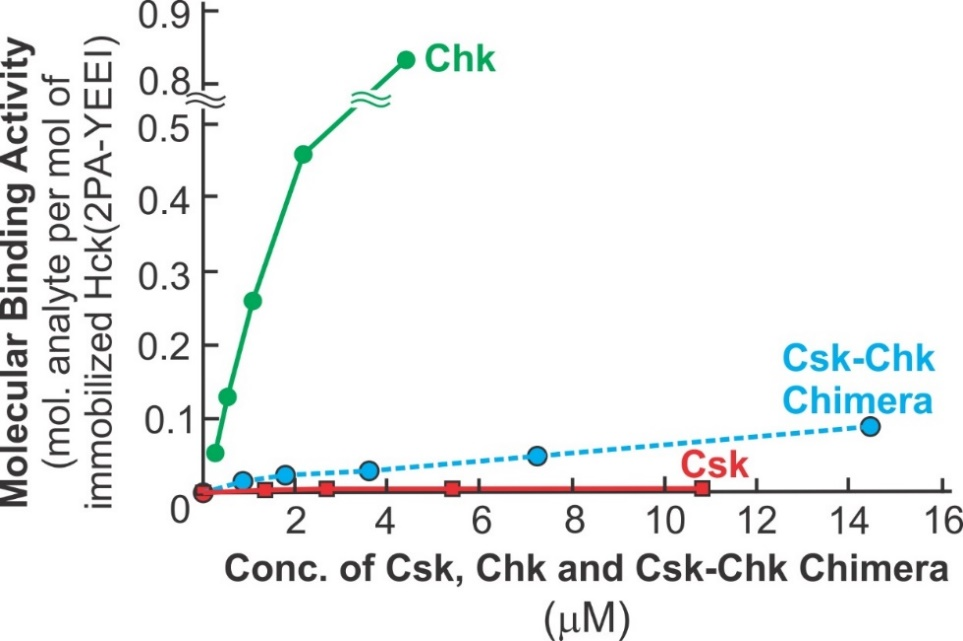

Supplement: Supplementary file 10 — Comparison of the molecular binding activities (M.B.A.) of Chk, Csk-Chk chimera and Csk. The M.B.A. values of Chk, Csk-Chk Chimera and Csk were plotted against at the designated concentrations used in the surface plasmon resonance spectroscopic analysis of the kinetics of their binding to the immobilised Hck (2PA-YEEI) (Figs. 4 and 5). Calculation of the M.B.A. values was presented in Additional file 8: Table S1. (TIFF 309 kb) [file 12964_2017_186_MOESM10_ESM.tif]

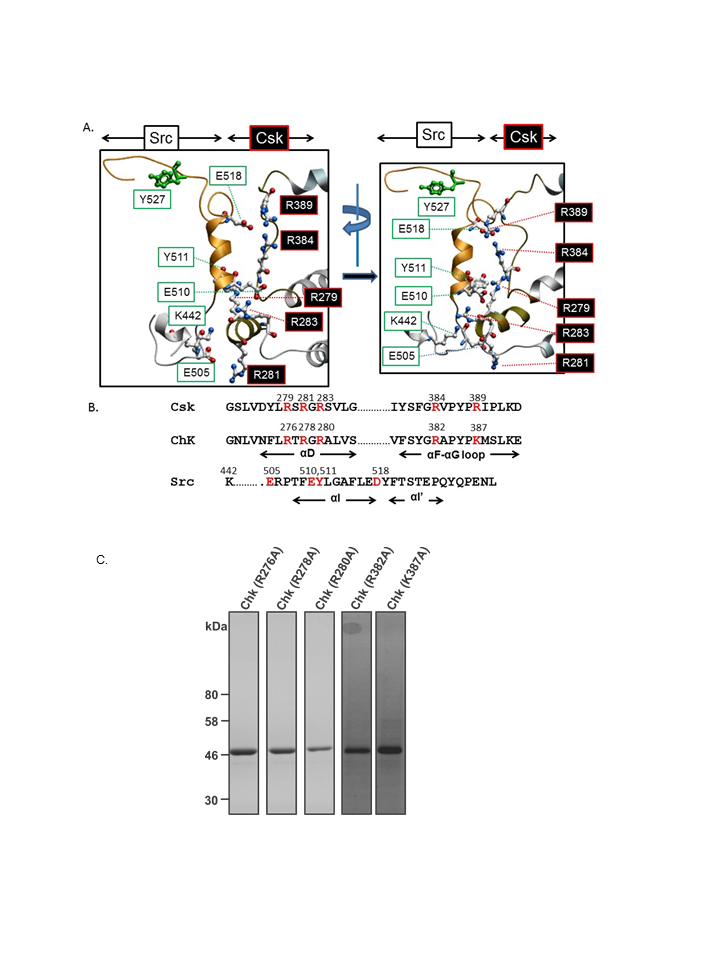

Supplement: Supplementary file 11 — Key residues involved in electrostatic and hydrophobic interactions at the Src-Csk interface and recombinant Chk mutants generated. A. The five conserved basic residues in the αD-helix and αF-αG loop of Csk interact electrostatically and hydrophobically with Lys-442 and acidic and hydrophobic residues in the αH-αI loop, αI and αI’ helices of Src. The image was generated by Molsoft L.L.C. using the coordinates of the structure of Csk/Src complex (PDB ID: 3D7T). The C-terminal tail tyrosine (Tyr-527) is shown in green. B. Alignment showing the five conserved basic residues in the Csk and Chk sequences. Lys-442 and the C-terminal tail sequence of Src at the Csk/Src interface are shown. The key residues participating in direct interactions with Csk are in red. C. Coomassie blue-stained gels showing the purity of purified Chk and its mutants used in this study. (TIFF 279 kb) [file 12964_2017_186_MOESM11_ESM.tif]

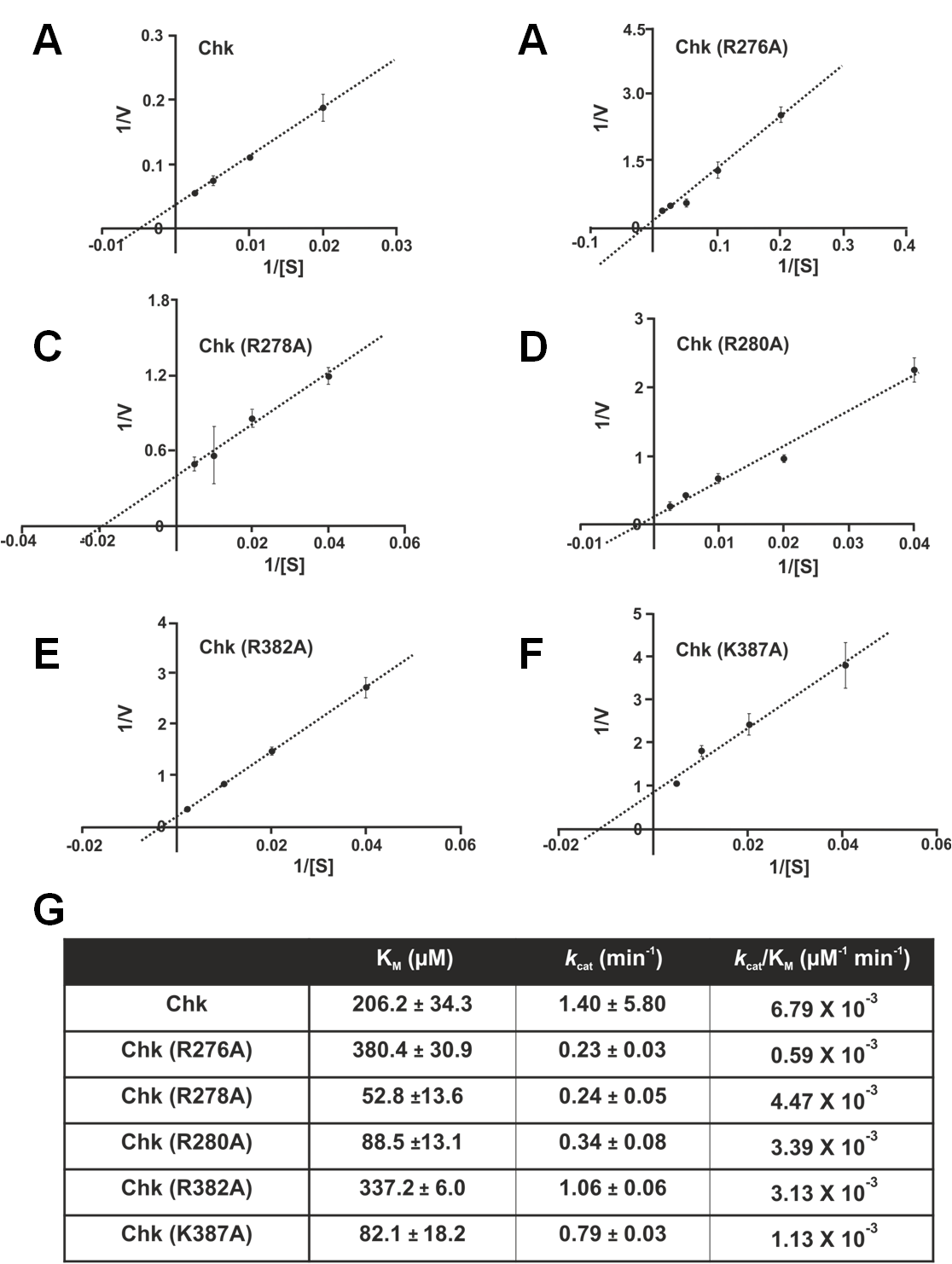

Supplement: Supplementary file 12 — Kinetic analysis of phosphorylation of Chk/Csk-optimal peptide by Chk (R276A), Chk (R278A), Chk (R280A), Chk (R382A) and Chk (K387A). A-F. Lineweaver-Burk plots of wild-type Chk (A), Chk (R276A) (B), Chk (R278A) (C), Chk (R280A) (D), Chk (R382A) (E) and Chk (K387A) (F). G. Kinetic parameters of the catalytic activity of Chk and its mutants including the Michaelis-Menten constant (KM), the catalytic constant (k cat) and the specificity constant (k cat/KM). (TIFF 463 kb) [file 12964_2017_186_MOESM12_ESM.tif]

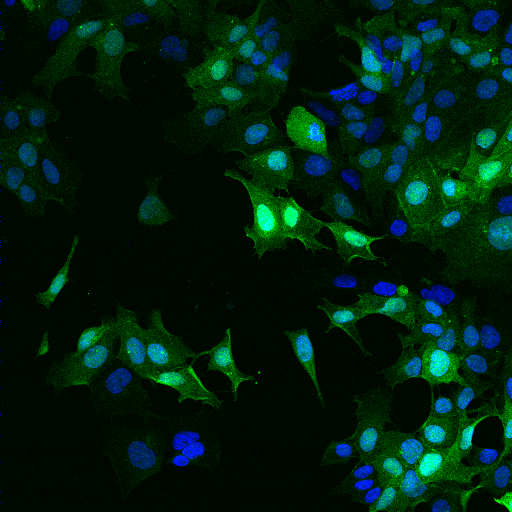

Supplement: Supplementary file 13 — Immunofluorescence analysis of transduced DLD1 cells expressing the recombinant CHK-GFP. A close-up image of one of the “merge” panels in Fig. 7b. Green: CHK-GFP; blue: DAPI stain. (TIFF 302 kb) [file 12964_2017_186_MOESM13_ESM.tif]

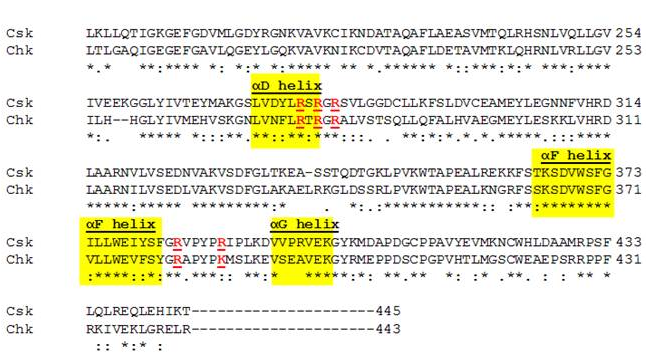

Supplement: Supplementary file 15 — Alignment of the sequences of Csk and Chk kinase domains. Arg-279, Arg-281 and Arg-283 of αD helix and the αD/αE loop, and Arg-384 and Arg-389 of αF/αG loop of Csk and the homologous basic residues in Chk are shown in red. (TIFF 564 kb) [file 12964_2017_186_MOESM15_ESM.tif]
