# Supplementary material for: Csk-homologous kinase (Chk) is an efficient inhibitor of Src-family kinases but a poor catalyst of phosphorylation of their C-terminal regulatory tyrosine
Source: Cell Commun Signal. 2017 Aug 7;15:29. doi: 10.1186/s12964-017-0186-x (PMC5547543; doi:10.1186/s12964-017-0186-x)
Supplement: Supplementary file 8 — Determination of the molecular binding activity of Chk, Csk and Csk-Chk Chimera to immobilized Hck(2PA-YEEI) by surface plasmon spectroscopy. (PDF 447 kb) [file 12964_2017_186_MOESM8_ESM.pdf]

Csk concentration (µM)	Hck(2PA-YEEI) MW	Csk MW	Immobilised Hck(2PA-YEEI) (RU)	Analyte (Csk) Response	Molecular Binding Activity (analytes per immobilised ligands)
10.8	52356.75	50704.43	6199	20	0.017
5.4	52356.75	50704.43	6199	15.1	0.014
2.7	52356.75	50704.43	6199	10.5	0.01
1.35	52356.75	50704.43	6199	6.5	0.007

Csk-Chk chimera concentration (µM)	Hck(2PA-YEEI) MW	Csk-Chk chimera MW	Immobilised Hck(2PA-YEEI) (RU)	Analyte (Csk/Chk chimera) response	Molecular Binding Activity (analytes per immobilised ligands)
14.45	52356.75	53913.87	6199	557	0.087
7.23	52356.75	53913.87	6199	301	0.047
3.61	52356.75	53913.87	6199	184	0.029
1.81	52356.75	53913.87	6199	150	0.023
0.9	52356.75	53913.87	6199	80	0.013

Table S1 Determination of the molecular binding activity of Chk, Csk and Csk-Chk Chimera to immobilized Hck(2PA-YEEI) by surface plasmon spectroscopy

Hck(2PA-YEEI) was immobilized on the pre-activated sensor chip to give 6199 response unit (RU). Chk and Csk of the designated concentrations were used as the analytes for measurement of their binding to the immobilized Hck(2PA-YEEI). The amount of Csk or Chk bound to the immobilized Hck(2PA-YEEI) was expressed as response units (see columns labelled "Analyte (Chk) Response" and "Analyte (Csk) Response"). The molecular binding activity, which is a measure of the amount of analyte molecule bound per molecule of Hck(2PA-YEEI) immobilized on the sensor chip is calculated by the following formula:

$$\text{Molecular Binding Activity} = \frac{[\text{Analyte (Csk or Chk) response}] \times [\text{Ligand (Hck(2PA-YEEI) M.W.}]}{[\text{Amount of immobilized ligand (Hck(2PA-YEEI)}] \times (\text{Analyte (Csk or Chk) M.W.})}$$

From the binding sensorgrams of Chk, Csk and Csk-Chk chimera bound to the immobilized Hck(2PA-YEEI) (Figures 4C, 4D and 5C), the kinetic parameters of interaction of Chk and Csk with Hck(2PA-YEEI) were determined and presented in Figures 4E and 5D.
