# Supplementary material for: Csk-homologous kinase (Chk) is an efficient inhibitor of Src-family kinases but a poor catalyst of phosphorylation of their C-terminal regulatory tyrosine
Source: Cell Commun Signal. 2017 Aug 7;15:29. doi: 10.1186/s12964-017-0186-x (PMC5547543; doi:10.1186/s12964-017-0186-x)
Supplement: Supplementary file 14 — Identification of tryptic fragments derived from Src co- immunoprecipitated with Chk-GFP from the DLD-1-Chk-GFP cell lysate. Proteins immunoprecipitated from lysate of the DLD-1-Chk-GFP cells and that of the DLD-1-GFP cells (Control) were processed and digested with trypsin as described previously by Ang and Nice [56]. The tryptic fragments were analysed by LC-MS/MS as described in the experimental procedures. The SRC peptides identified by LC MS/MS and using the SEQUEST search engine are listed. A total of 4 different peptides were identified with high confidence at 1% FDR. The identify of these peptides were further validated with the Percolator algorithm for discrimination between correct and incorrect spectrum identifications as described by Kall, et al [84]. These fragments were not detected in the anti-GFP immunoprecipitate of the DLD-1-GFP cell lysate (Control lysate), suggesting that Src was specifically bound to Chk-GFP in the DLD-1-Chk-GFP cells. (PDF 93 kb) [file 12964_2017_186_MOESM14_ESM.pdf]

Sequence in Protein	Positions in human Src	Modifications	XCorr Sequest HT	Percolator q-Value Sequest HT	Percolator PEP Sequest HT
[R]LLLNAENPR[G]	[170-178]		2.28	0.000192	0.00773
[R]AANILVGENLVCK[V]	[398-410]	1×Carbamidomethyl [C12]	2.35	0.000192	0.00416
[K]VADFGLAR[L]	[411-418]		1.91	0.000672	0.0235
[R]TQFNSLQQLVAYYSK[H]	[227-241]		3.2	0.000672	0.0146

Table S2 Identification of tryptic fragments derived from Src co-immunoprecipitated with Chk-GFP from the DLD-1-Chk-GFP cell lysate

Proteins immunoprecipitated from lysate of the DLD-1-Chk-GFP cells and that of the DLD-1-GFP cells (Control) were processed and digested with trypsin as described previously by Ang and Nice [56]. The tryptic fragments were analysed by LC-MS/MS as described in the experimental procedures. The SRC peptides identified by LC MS/MS and using the SEQUEST search engine are listed. A total of 4 different peptides were identified with high confidence at 1% FDR. The identify of these peptides were further validated with the Percolator algorithm for discrimination between correct and incorrect spectrum identifications as described by Kall, *et al* [84]. These fragments were not detected in the anti-GFP immunoprecipitate of the DLD-1-GFP cell lysate (Control lysate), suggesting that Src was specifically bound to Chk-GFP in the DLD-1-Chk-GFP cells.
